# Energy‐Based Phase‐Locking State Analysis in Brain State Identification

**DOI:** 10.1002/hbm.70558

**Published:** 2026-06-05

**Authors:** Chenfei Ye, Ziyan Deng, Shiqing Cong, Chen Ran, Tao Gong, Shoulin Huang, Ting Ma

**Affiliations:** ^1^ School of Biomedical Engineering, Harbin Institute of Technology (Shenzhen) Shenzhen China; ^2^ School of Information Science and Technology, Harbin Institute of Technology (Shenzhen) Shenzhen China; ^3^ Department of Radiology Shandong Provincial Hospital Affiliated to Shandong First Medical University Jinan China; ^4^ School of Electronic and Information Engineering, Guangxi Normal University Guilin China

**Keywords:** brain network dynamics, dynamic functional connectivity, energy landscape, fMRI, maximum entropy model, neural synchronization

## Abstract

The human brain exhibits inherent multistability, with Energy Landscape Analysis (ELA) providing effective frameworks for investigating this property through BOLD signals. However, traditional amplitude‐based approaches fundamentally neglect critical phase synchronization dynamics that mediate large‐scale neural coordination, while existing phase‐based methods like Leading Eigenvector Dynamic Analysis (LEiDA) lack thermodynamic formalism for state stability quantification. Here, we introduce Energy‐based Phase‐Locking State Analysis (EPLSA), a transformative computational framework that synergistically integrates instantaneous phase‐coupling dynamics with rigorous energy landscape principles, addressing fundamental limitations of conventional methodologies. Comprehensive validation across two independent neuroimaging datasets (HCP and Natural Sleep) demonstrated EPLSA's marked superiority over LEiDA and conventional ELA in terms of test–retest reliability, task‐specific brain state differentiation, and individual‐level classification performance. To demonstrate the physiological and clinical utility of the proposed method, sleep–wake analysis was performed to reveal EPLSA's enhanced sensitivity to consciousness state transitions, identifying decreased primary state occupancy and increased minor state prevalence during sleep, with significantly reduced direct transition probabilities. Furthermore, application to patients with Alzheimer's disease using the OASIS‐3 dataset identified shortened dwell time and occurrence frequency for the frontoparietal control network‐default mode network (FPCN‐DMN) co‐activation state, and prolonged dwell time and occurrence frequency for the visual network‐limbic network (VIS‐LMN) co‐activation state, with these metrics significantly correlating with cognitive impairment. By unifying phase‐coupling and thermodynamic principles, EPLSA provides novel insights into neurodynamic mechanisms across cognitive tasks, consciousness states, and neurodegenerative conditions, offering a transformative analytical tool for investigating brain function in health and disease with particular promise for early detection and monitoring of neurological disorders.

## Graphical abstract

EPLSA constructs an energy landscape from BOLD phase synchrony, achieving superior brain state classification and providing novel stability metrics. It demonstrates strong clinical translatability in characterizing sleep–wake transitions and Alzheimer's disease.

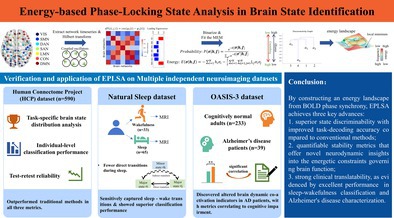

## 1 Introduction

The systematic investigation of dynamic properties in brain functional activity through multidimensional neuroimaging time‐series analysis, particularly functional magnetic resonance imaging (fMRI), represents a fundamental endeavor in contemporary neuroscience. A critical methodological paradigm in this domain involves the inference of temporal evolution patterns within discrete brain states, wherein each state may correspond to distinct functional network configurations (Allen et al. 2014; Baker et al. 2014; Calhoun et al. 2014; Nielsen et al. 2018; Ryali et al. 2016; Taghia et al. 2017) or specific spatial activation patterns (Ezaki et al. 2021; Rashid et al. 2014). Contemporary neuroimaging evidence demonstrates that the resting brain cannot be characterized as a singular static entity, but rather exhibits complex spatiotemporal dynamics characterized by transitions among multiple stable states (Cabral et al. 2014; Deco and Jirsa 2012; Deco et al. 2015; Freyer et al. 2011; Freyer et al. 2012; Tognoli and Kelso 2014). Within this framework, a stable state is operationally defined as a self‐sustaining activity pattern that persists over finite temporal scales while demonstrating resilience to perturbations. Systems exhibiting multiple coexisting stable states that can undergo transitions in response to stochastic noise or intrinsic perturbations are characterized as multistable systems (Kelso 2012). Consequently, the human brain can be conceptualized as a fundamentally multistable system.

The conceptual framework of multistability in brain dynamics has been effectively investigated through energy landscape analysis (ELA), which provides a principled theoretical foundation for characterizing brain dynamics as the movement of a stochastic particle constrained within an energy landscape derived from empirical data (Ezaki et al. 2017; Watanabe, Hirose, et al. 2014; Watanabe, Masuda, et al. 2014). This approach enables intuitive interpretations through quantification of estimated energy landscapes: local energy minima correspond to specific spatial activity patterns that define discrete brain states, while high energy barriers between minima indicate increased difficulty for brain dynamics to transition between these states. The demonstrated efficacy of energy landscape analysis is evidenced by its robust associations with behavioral measures across diverse domains, including bistable visual perception (Watanabe 2021; Watanabe, Masuda, et al. 2014), executive function (Kang et al. 2021), fluid intelligence (Ezaki et al. 2020), and healthy aging (Ezaki et al. 2018), as well as its clinical utility in characterizing pathological conditions including autism spectrum disorders (Watanabe and Rees 2017), Alzheimer disease (Klepl et al. 2021), schizophrenia (Allen et al. 2021; Braun et al. 2021), attention deficit hyperactivity disorder (Udall and Choa 2022) and epilepsy (Krzeminski et al. 2020). However, a fundamental limitation of conventional ELA approaches is their exclusive reliance on BOLD signal amplitude information, thereby neglecting critical coupling coordination between neural populations and brain regions—dynamics that represent essential spatiotemporal interactions underlying brain function.

Neural synchronization, defined as the coordination in states between two or more systems attributable to their interaction or coupling, represents a fundamental mechanism of brain organization. Phase synchronization (PS) methods have been developed to quantify synchrony levels between time series from different brain regions of interest (ROIs) (Glerean et al. 2012; Pedersen et al. 2017, 2018), wherein the instantaneous phase of each time series is computed through Hilbert transform application, enabling evaluation of phase differences between paired time series. Building upon this foundation, Leading Eigenvector Dynamic Analysis (LEiDA) represents a significant methodological advancement based on spatial coherence analysis of BOLD phase signals (Cabral et al. 2017). LEiDA employs instantaneous phase synchronization methods to generate phase alignment matrices, subsequently extracting the leading eigenvector to characterize the dominant coupling mode at each temporal point. Clustering of these leading eigenvectors across all time points enables characterization of recurrent spatial patterns corresponding to distinct brain states. LEiDA has demonstrated substantial contributions across diverse functional brain paradigms, yielding insights into sleep–wake transitions (Deco et al. 2019), psychedelic drug actions (Lord et al. 2019; Olsen et al. 2022), neurodevelopment (França et al. 2022), schizophrenia (Hancock et al. 2023), and depression (Figueroa et al. 2019; Martínez et al. 2020). Importantly, LEiDA addresses the dimensionality challenge inherent in dynamic functional connectivity (dFC) methods through leading eigenvector extraction, enabling characterization of dynamic brain functions based on BOLD phase coherence patterns that more sensitively capture instantaneous synchronization between neural populations.

Despite these notable advantages, LEiDA possesses inherent methodological limitations that constrain its analytical capabilities. This clustering‐based approach is conceptually aligned with the “ghost attractors” framework proposed by Vohryzek et al. (2020), which describes weakly stable, transient phase‐locking configurations that recurrently emerge in spontaneous brain activity. However, while LEiDA effectively captures the occurrence of these functional excursions, it lacks a formal theoretical framework for quantifying the thermodynamic stability or the energy barriers that govern transitions between these configurations. The absence of thermodynamic principles in LEiDA precludes direct quantification of state persistence, transition probabilities, and the energetic cost associated with network reconfiguration—critical parameters for understanding brain dynamics from a systems perspective. Furthermore, LEiDA's clustering‐based approach, while effective for state identification, does not provide mechanistic insights into the underlying dynamical constraints that determine state accessibility and stability. These limitations become particularly pronounced when investigating clinical populations or altered states of consciousness, where understanding the energetic landscape of brain states is crucial for characterizing pathophysiological mechanisms. Therefore, we hypothesize that integrating phase coherence features from LEiDA into the energy landscape framework will more directly reflect dynamic constraints governing brain state transitions, such as energy barriers associated with phase synchronization patterns, thereby enhancing analytical capabilities for investigating brain state stability and transition mechanisms.

**FIGURE 1:**
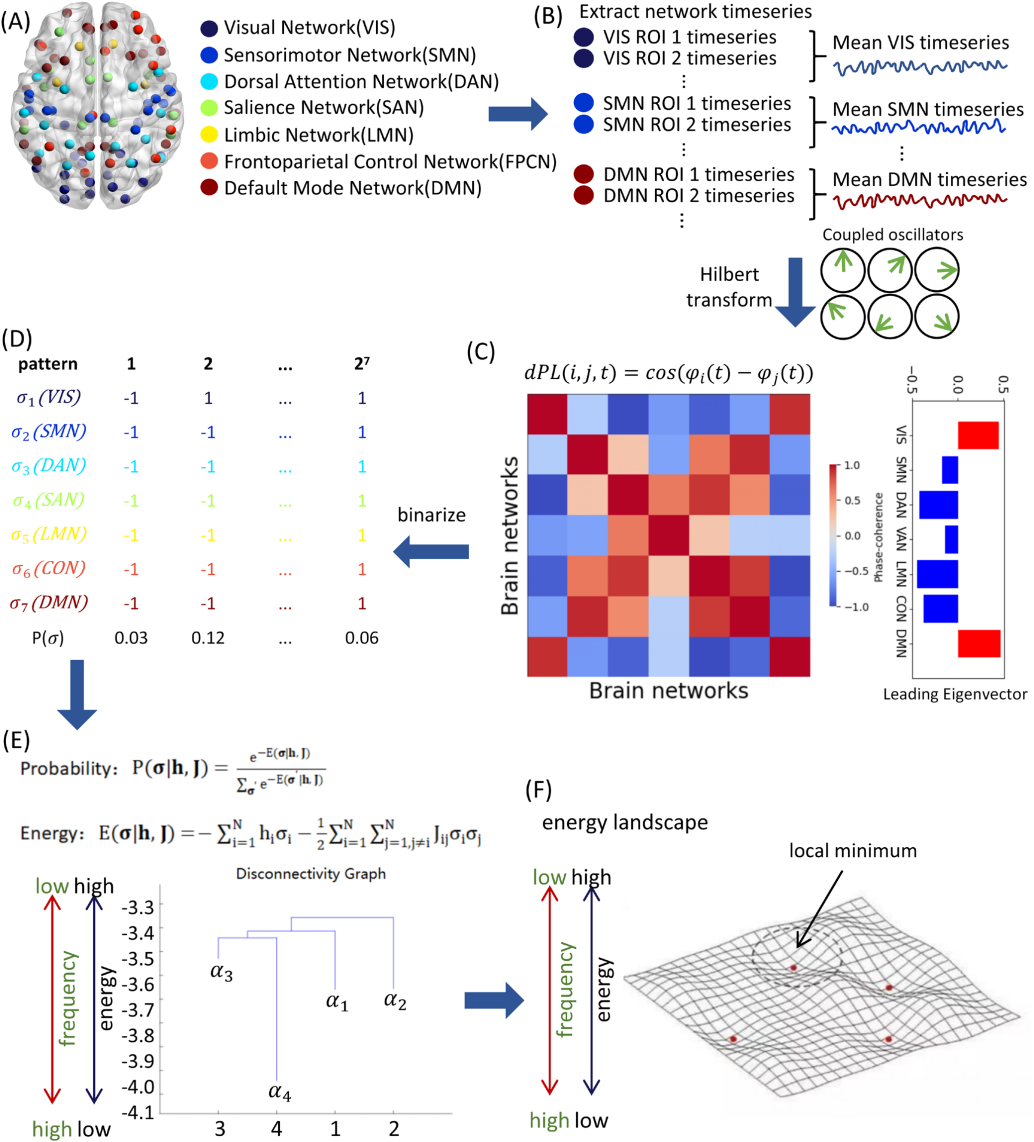
Methodological framework and computational pipeline of Energy‐based Phase‐Locking State Analysis (EPLSA). (A) Parcellation of brain regions into seven canonical functional networks based on established neuroanatomical atlases. (B) Extraction of network‐averaged BOLD time series for each functional network. (C) Phase extraction via Hilbert transform and computation of dynamic phase‐locking (dPL) matrices at each time point, followed by leading eigenvector extraction. (D) Binarization of leading eigenvectors into active (+1) and inactive (−1) states, with subsequent calculation of normalized occurrence frequencies for each binary activity pattern. (E) Maximum entropy modeling (MEM) of empirical activity pattern distributions across 2^*N*^ possible configurations, yielding energy values for each pattern and construction of disconnectivity diagrams representing energy landscape topology. (F) Comprehensive energy landscape visualization depicting attractor basins and transition pathways. Methodological framework adapted from Ezaki et al. (2017).

The present study introduces a computational approach termed Energy‐based Phase‐Locking State Analysis (EPLSA), which constructs energy landscapes based on leading eigenvectors of instantaneous phase alignment matrices. This methodology simultaneously incorporates phase coupling information while circumventing dimensionality challenges associated with conventional dFC approaches and, crucially, extends LEiDA by introducing formal energy metrics derived from statistical physics principles (see Figure 1 for details). We systematically evaluated EPLSA performance against established methods (LEiDA and ELA) using multiple validation criteria, including test–retest reliability, task‐specific brain state differentiation, and individual‐level classification performance. To further establish the robustness and broad applicability of EPLSA, we applied this approach to natural sleep datasets to assess its capacity for distinguishing wakefulness and sleep states. Finally, we employed EPLSA to characterize brain dynamic differences between Alzheimer's disease (AD) patients and healthy controls, thereby confirming the potential clinical applications of this approach.

## 2 Materials and Methods

### 2.1 Study Populations and Imaging Protocols

We analyzed three independent neuroimaging datasets to evaluate the robustness and broad applicability of our methodological approach.

#### 2.1.1 Human Connectome Project (HCP) Dataset

A cohort of 590 healthy adults underwent (280 males, 310 females; age range: 22–36 years; mean ± SD = 28.8 ± 3.7 years) whole‐brain fMRI as part of the S1200 Human Connectome Project (Van Essen et al. 2013; Barch et al. 2013). The experimental protocol comprised seven cognitive tasks (Motor, Emotion, Gambling, Language, Relational, Social, Working memory), as well as resting‐state conditions. Each task was performed twice with counterbalanced phase‐encoding directions: right‐to‐left (RL) and left‐to‐right (LR).

#### 2.1.2 Natural Sleep Dataset

Thirty‐three healthy participants (17 males, 16 females; age range: 18–29 years; mean ± SD = 22.1 ± 3.2 years) were recruited for simultaneous fMRI‐EEG recording during natural sleep (Gu et al. 2022, 2023). Sleep staging was performed using standard polysomnography, identifying wake and sleep stages (N1, N2, N3). The final analyzed cohort included 33 participants who reached N1 sleep; 29 who reached N2 sleep, and three who achieved N3 sleep. All procedures were approved by the Penn State Ethics Committee.

#### 2.1.3 OASIS‐3 Dataset

OASIS‐3 is a compilation of MRI and PET imaging and related clinical data for 1098 participants collected across several ongoing studies at the Washington University Knight Alzheimer Disease Research Center over 15 years. Participants included 605 cognitively normal adults and 493 individuals at various stages of cognitive decline, ranging in age from 42 to 95 years (LaMontagne et al. 2019). All participants met OASIS‐3's basic requirements, including completion of at least one Uniform Data Set (UDS)‐compliant clinical assessment, passage of imaging quality control, compliance with anonymization protocols, and provision of IRB‐approved informed consent. Detailed inclusion criteria for the Healthy Control (HC) group and the AD‐related Cognitive Impairment group, including cognitive status, amyloid levels (defined by tracer‐specific Centiloid thresholds), and age matching, are provided in Supporting Information File S1 (Su et al. 2019).

### 2.2 Image Preprocessing

The three datasets employed distinct, well‐established preprocessing protocols tailored to their specific acquisition parameters and data quality considerations. Applying the EPLSA framework across these varied pipelines demonstrates its robustness and generalizability.

#### 2.2.1 HCP Dataset

The brains were normalized to fslr32k by MSM‐AII registration in 100 regions (Schaefer et al. 2018). CompCor utilized five principal components from the ventricle and white matter mask to eliminate interference signals from the time series through regression. Additionally, 12 non‐trend motion estimates provided by the Human Connectome Project were retrieved from regional time series. The average global signal was removed, and then the time series was bandpass filtered within the range of 0.009–0.08 Hz. Global signal regression (GSR) was applied to mitigate the potential influence of non‐neuronal physiological noise (e.g., respiratory and cardiac fluctuations) and motion artifacts on the BOLD signal, which can significantly affect functional connectivity estimates and induce spurious correlations (Murphy et al. 2009; Power et al. 2015). While acknowledging that GSR may also attenuate neurobiologically meaningful global synchronization (Cabral et al. 2017), this step was implemented to enhance the specificity of network interactions under investigation, following preprocessing pipelines optimized for minimizing false behavioral associations in the HCP dataset (Siegel et al. 2017). Frames with interframe displacement greater than 0.2 mm or derivative root mean square (DVARS) greater than 75 were subsequently removed as outliers. We filtered out sessions consisting of more than 50% of abnormal frames, and we only analyzed the data of all scanned subjects remaining after filtering, leaving 590 individuals. The processing pipeline employed here was previously considered ideal for eliminating the false relationship between neuro dynamics and behavior (Siegel et al. 2017). After the signal processing, the activity of each brain region was discretized in time, generating a time series of neural activity.

#### 2.2.2 Natural Sleep Dataset

The preprocessing of fMRI data adhered to a comprehensive protocol established in previous studies (Huang, Tarnal, et al. 2021a; Huang, Tarnal, et al. 2021b; Huang et al. 2020). The AFNI software package (linux_ubuntu_16_64; http://afni.nimh.nih.gov/) was utilized. Steps include: (1) Slice timing correction; (2) Correction and adjustment of rigid body head motion; (3) Scrubbed frame by frame to solve the head motion artifact; (4) Co‐registration with T1‐weighted anatomical images; (5) Spatial normalization and resampled to 3 × 3 × 3 mm; (6) Band‐pass filtering to 0.01–0.1 Hz while regressing out unwanted noise; (7) Spatial smoothing with 6 mm full width at half maximum isotropic Gaussian kernel; (8) Temporal normalization to zero mean and unit variance. The cortex was defined by 400 cortical regions, according to a well‐established cortical segmentation scheme (Schaefer et al. 2018; Yeo et al. 2011). Subcortical was defined by 50 regions, which are derived from the Subcortical Atlas (Tian et al. 2020). fMRI time processes are extracted from these 450 regions of interest (ROIs).

#### 2.2.3 OASIS‐3 Dataset

The preprocessing of MRI data in the OASIS‐3 dataset followed a standardized protocol to ensure high data quality and consistency. The FreeSurfer software suite (version 7.2.0; https://surfer.nmr.mgh.harvard.edu/) was employed for cortical reconstruction and volumetric segmentation, while FSL (FMRIB Software Library; version 6.0.4; https://fsl.fmrib.ox.ac.uk/fsl/fslwiki/) was utilized for additional processing steps. The specific preprocessing pipeline included the following steps: (1) Motion Correction and Spatial Registration; (2) Bias Field Correction; (3) Skull Stripping; (4) Cortical Reconstruction and Segmentation; (5) Spatial Normalization; (6) Smoothing. The cortical regions were parcellated into 400 distinct ROIs using the Schaefer atlas (Schaefer et al. 2018), which provides a well‐established framework for cortical segmentation.

### 2.3 Energy‐Based Phase‐Locking State Analysis (EPLSA)

We adopted network‐level analysis based on seven canonical functional networks rather than ROI‐level analysis for three key reasons. First, energy landscape analysis and maximum entropy model fitting suffer from the curse of dimensionality: the state space scales as $2^{N}$, where *N* is the number of units. With *N* = 7 networks, the state space contains only 128 configurations, enabling stable and reliable parameter estimation. By contrast, ROI‐level parcellations (e.g., 400 ROIs) yield a computationally intractable state space and severe overfitting. Second, large‐scale functional networks represent biologically meaningful functional units whose reorganization is central to consciousness transitions, cognitive tasks, and neurodegenerative pathophysiology. Network‐level dynamics thus offer direct mechanistic and interpretability advantages over fine‐grained ROI signals. Third, even though our benchmark comparisons included ROI‐level LEiDA (LEiDA‐roi), network‐based EPLSA achieved consistently superior performance in state discrimination, classification, and reliability, indicating that the integration of phase‐locking dynamics and energy landscape principles captures core brain state properties beyond spatial resolution.

#### 2.3.1 Leading Eigenvector Extraction and Binarization

Network BOLD timeseries were extracted by mapping ROIs to seven classical resting‐state networks (Yeo et al. 2011) (see Figure 1A,B). Then we obtained the phase $\varphi_{j}\left(t\right)$ for each region in time with the Hilbert transform. For a given real signal *s*(*t*), we built a complex signal *z*(*t*) given by:

(1) $$z\left(t\right)=s\left(t\right)+\mathrm{iH}\left[s\left(t\right)\right]$$

In which $H\left[s\left(t\right)\right]$ represented a Hilbert transform applied to the real signal *s(t)* and is defined below, with p.v. consisting of Cauchy principal value:

(2) $$\begin{array}{c} H\left[s\left(t\right)\right]=p.v.\int_{-\infty }^{\infty }\frac{s\left(t-\tau \right)}{\mathrm{\pi t}}\mathrm{dt} \end{array}$$

The instantaneous phase $\varphi_{j}\left(t\right)$ for each parcel was calculated directly from *z*(*t*):

(3) $$\begin{array}{c} \varphi_{j}\left(t\right)=\arctan\left(\frac{H\left[s\left(t\right)\right]}{s\left(t\right)}\right) \end{array}$$

Phase synchronization between brain regions *i* and *j* at each time *t* was quantified using a dynamic Phase‐Locking matrix:

(4) $$\begin{array}{c} \mathrm{dPL}\left(i,j,t\right)=\cos\left(\varphi_{i}\left(t\right)-\varphi_{j}\left(t\right)\right) \end{array}$$

This yielded a symmetric NxNxT tensor (−1 ≤ dPL ≤ 1) representing instantaneous phase relationships.

Following the LEiDA framework (Cabral et al. 2017; Lord et al. 2019), we extracted the leading eigenvector from each dPL(*t*) matrix to obtain a lower‐dimensional representation of whole‐brain phase coherence patterns. The leading eigenvector captures the dominant mode of phase synchronization across brain networks at each time point.

Network activity patterns were subsequently binarized based on leading eigenvector values, yielding seven binary time series representing network states (+1 for active, −1 for inactive). The binarization threshold is mathematically fixed at zero, determined by the sign of the eigenvector components, which ensures that the resulting states reflect the intrinsic bipartite phase‐synchrony structure without arbitrary parameter selection. This binarization procedure balanced active and inactive states for each network, reducing overfitting risk in subsequent analyses (Bishop and Nasrabadi 2006).

#### 2.3.2 Maximum Entropy Model Fitting

As a preparation for the following energy‐landscape analysis, we fitted a pairwise Maximum entropy model (MEM) to the seven binary time series data in essentially the same manner as in previous studies (Ezaki et al. 2017; Watanabe et al. 2013). MEM is a probabilistic framework that infers the most unbiased (maximum entropy) distribution of neural activity patterns while preserving empirically observed pairwise correlations between brain regions or networks. By constraining only the first‐ and second‐order moments of the data, MEM avoids overfitting while capturing the essential statistical structure of large‐scale brain dynamics.

First, every network activity pattern at time *t* is given by an *N*‐dimensional vector $\sigma \equiv \left(\sigma_{1},\ldots ,\sigma_{N}\right)\in {\left\{-1,1\right\}}^{N}$, where $\sigma_{i}$ represents the binary activity of network *i* at time *t* (i.e., +1 for active, −1 for inactive), and *N* is the number of the networks (*N* = 7 here, Figure 1A). Note that there are $2^{N}$ possible activity patterns in total. Then, we calculate the relative frequency with which each activity pattern is visited, $P_{\text{empirical}}\left(\sigma \right)$ (Figure 1d). To $P_{\text{empirical}}\left(\sigma \right)$, we fit the Boltzmann distribution given by:

(5) $$\begin{array}{c} P\left(\sigma |h,J\right)=\frac{e^{-E\left(\sigma |h,J\right)}}{\sum_{\sigma^{\prime }}e^{-E\left(\sigma^{\prime }|h,J\right)}} \end{array}$$

In this probabilistic framework, the energy value represents the negative log‐probability of a state, such that brain activity patterns occurring more frequently in the empirical data are assigned lower energy values. This formulation reflects the higher thermodynamic stability of these states within the energy landscape.

(6) $$\begin{array}{c} E\left(\sigma |h,J\right)=-\sum_{i=1}^{N}h_{i}\sigma_{i}-\frac{1}{2}\sum_{i=1}^{N}\sum_{j=1,j\neq i}^{N}J_{\mathrm{ij}}\sigma_{i}\sigma_{j} \end{array}$$

Here, $h=\left\{h_{i}\right\}$ represents basal activity of network *i* and $J=\left\{J_{\mathrm{ij}}\right\}$ indicates a pairwise interaction between networks *i* and *j*, they are the parameters of the model. We assume $J_{\mathrm{ij}}$ = $J_{\mathrm{ji}}$ and $J_{\mathrm{ii}}=0$ (*i, j* = 1,⋯, *N*). The principle of maximum entropy imposes that we select ***h*** and ***J*** such that ${\left\langle \sigma_{i}\right\rangle }_{\text{empirical}}={\left\langle \sigma_{i}\right\rangle }_{\text{model}}$ and ${\left\langle \sigma_{i}\sigma_{j}\right\rangle }_{\text{empirical}}={\left\langle \sigma_{i}\sigma_{j}\right\rangle }_{\text{model}}$, where ${\left\langle \cdots \right\rangle }_{\text{empirical}}$ and ${\left\langle \cdots \right\rangle }_{\text{model}}$ represent the mean with respect to the empirical distribution and the model distribution, respectively.

#### 2.3.3 Energy Landscape Construction and State Identification

Once we have estimated the pairwise MEM, we construct a dendrogram referred to as a disconnectivity graph (Van Essen et al. 2013), as shown in Figure 1E. In the disconnectivity graph, a leaf (with a loose end open downwards) corresponds to an activity pattern σ that is a local minimum of the energy, that is, an activity pattern whose frequency is higher than any other activity pattern in the neighborhood of σ. The neighborhood of σ is defined as the set of the *N* activity patterns that are different from *σ* only at one ROI.

To examine hierarchal structures between the detected local minimum, we then constructed disconnectivity graphs as follows (Watanabe, Hirose, et al. 2014; Watanabe, Masuda, et al. 2014). (i) We prepared a so‐called hypercube graph, in which each node representing a brain activity pattern was adjacent to the *N* neighboring nodes. (ii) We set a threshold energy level, $E_{\mathrm{th}}$, at the largest energy value among the $2^{N}$ nodes. (iii) We removed the nodes whose energy values were $\geq E_{\mathrm{th}}$. (iv) We examined whether each pair of local minimum $\left\langle \alpha ↔\alpha^{\prime }\right\rangle$ was connected by a path in the reduced network. This connectivity information at each Eth determines their hierarchical relationship in the final disconnectivity graph. (v) We repeated steps (iii) and (iv) after moving $E_{\mathrm{th}}$ down to the next largest energy value. We ended up with a reduced network in which each local min was isolated. (vi) On the basis of the obtained results, we built a hierarchical tree whose leaves (i.e., terminal nodes down in the tree) represented the local minimum α and internal nodes indicated the branching points of different local minimum.

The attractive basin is a set of activity patterns. Each local minimum has a basin of attraction in the state space, *Ω*. Each activity pattern, denoted by σ, usually belongs to one of the attractive basins, which is determined as follows. (i) Unless σ is a local minimum, move to the neighboring activity pattern that has the smallest energy value. (ii) Repeat step (i) until a local minimum, denoted by α, is reached. We conclude that σ belongs to the attractive basin of α. (iii) Repeat steps (i) and (ii) for all the initial activity patterns σ∈Ω. And the basin size was defined as the fraction of the number of the brain activity patterns belonging to the basin. Using the information on the local minimums and attractive basins, the dynamics of the activity pattern are illustrated as the motion of a “ball” on the energy landscape, as schematically shown in Figure 1F as a hypothetical two‐dimensional landscape. The local minimums and energy barriers in Figure 1F correspond to those shown in the disconnectivity graph (Figure 1E). We refer to each of these attractive basins as a brain state. The dynamics of such states can be studied with three main metrics: fractional occupancy—which refers to the total proportion of time spent in a given state or probability of that state; dwell time—which consists of the average continuous time spent on each state; and transition probabilities between each state (Cabral et al. 2017; Lord et al. 2019).

### 2.4 Benchmark Methodologies

To systematically evaluate the performance of EPLSA in brain state identification, we conducted comprehensive comparative analyses against established methodological approaches, including two variants of LEiDA and traditional Energy Landscape Analysis (ELA). The comparative framework was designed to assess methodological robustness across multiple analytical dimensions.

These two LEiDA variants were implemented to capture brain dynamics at different spatial scales and organizational levels. The first variant, LEiDA‐roi, applied the analysis framework directly to individual brain region time series, enabling the identification of dynamic states at high spatial resolution. This approach preserved the granular temporal patterns unique to each brain region, providing detailed characterization of local neural dynamics. The second variant, LEiDA‐net, operated on network‐averaged time series, where signals from anatomically defined brain regions were aggregated within predefined functional networks. This coarse‐graining procedure generated a more integrative representation of brain activity, specifically designed to elucidate collective network dynamics and inter‐network interactions governing state transitions.

Besides, we implemented traditional ELA using amplitude‐based binarization of BOLD signals. This established approach employs MEM to capture brain state dynamics through quantification of energy barriers and identification of local minima in the energy landscape. The methodological details of these benchmark approaches are comprehensively documented in Supporting Information Figure S1 and S2.

### 2.5 Comparative Methodology Evaluation

Comprehensive comparative assessment of methodological performance was conducted across four analytical approaches (EPLSA, LEiDA‐roi, LEiDA‐net, and traditional ELA) spanning three critical evaluation domains: (1) test–retest reliability, (2) task‐specific brain state differentiation, and (3) individual‐level classification performance:

- *Test–retest reliability assessment*: Brain state reliability was quantified using the Mean Nearest Rank (MNR) discriminability index, a nonparametric multivariate metric that evaluates the consistency of within‐subject measurements relative to between‐subject variability (Bridgeford et al. 2020). This discriminability measure assesses two fundamental properties: consistency, where repeated measurements from the same subject under identical conditions (i.e., scanning with LR and RL phase‐encoding directions for HCP dataset) should exhibit minimal distance; and differentiability, where measurements from distinct subjects should be readily distinguishable. The discriminability index specifically quantifies how consistently within‐subject distances remain smaller than between‐subject distances. The calculation pipeline was implemented as follows:
  1. *Distance matrix construction*: Brain state data were represented as k×k functional connectivity matrices (where k = number of brain regions). For n total measurements (across all subjects and scan sessions), the 3D graph data (shape: k×k×n) was reshaped to a 2D array ($n\times k^{2}$). Pairwise Euclidean distances between all measurements were computed using Euclidean distance metric, generating an n×n symmetric distance matrix Dwhere $D_{i,j}$ denotes the Euclidean distance between the *i*‐th and *j*‐th measurements.
  2. *Within‐subject rank computation*: For each within‐subject measurement pair (*i*, *j*), all distances belonging to the same subject as the current measurement were set to infinity to exclude within‐subject bias. The rank of the within‐subject distance *D*_*i*,*j*_ was calculated as follows:
(7) $$\begin{array}{c} r_{i,j}=1-\frac{\sum \left(D_{i}<D_{i,j}\right)+0.5\times \sum \left(D_{i}=D_{i,j}\right)}{N-S} \end{array}$$
    where *N* = total number of measurements, *S* = number of repeated measurements for the target subject, $\sum \left(D_{i}<D_{i,j}\right)$ = count of between subject distances smaller than $D_{i,j}$, and $0.5\times \sum \left(D_{i}=D_{i,j}\right)$ = tie correction for equal distances.

The final MNR value was the mean of all valid *r*_*i*,*j*_ (excluding self‐pairs *i* = *j*).

- *Task‐specific brain state distribution analysis*: To evaluate the discriminative capacity of brain state probability distributions across distinct HCP cognitive conditions, we computed symmetrized Kullback–Leibler (KL) divergence between task‐specific state occupancy patterns. The divergence metric was formulated as:
(8) $$\begin{array}{c} \mathrm{KL}\left(P_{\text{task}1},P_{\text{task}2}\right)=0.5\left(\sum_{i}P_{\text{task}1}\left(i\right)\ln\frac{P_{\text{task}1}\left(i\right)}{P_{\text{task}2}\left(i\right)}+\sum_{i}P_{\text{task}2}\left(i\right)\ln\frac{P_{\text{task}2}\left(i\right)}{P_{\text{task}1}\left(i\right)}\right) \end{array}$$
  where $P_{\text{task}1}\left(i\right)$ and $P_{\text{task}2}\left(i\right)$ represent the occupancy probabilities of metastable state *i* for task conditions 1 and 2, respectively. This symmetrized formulation ensures mathematical equivalence regardless of comparison order while quantifying the divergence between probability distributions of brain state occupancy across all pairwise task combinations (rest plus seven cognitive tasks in HCP, yielding C_8_^2^ = 28 total comparisons).
- *Individual‐level classification performance*: Classification capability was evaluated through supervised machine learning approaches employing random forest algorithms with 10‐fold cross‐validation. Two distinct classification experiments were implemented: (1) rest‐versus‐task discrimination using the HCP dataset, where classifiers distinguished resting‐state from seven cognitive task conditions; and (2) sleep–wake state discrimination using the Natural Sleep dataset. Performance metrics encompassed accuracy (ACC), precision, recall, and F1‐score, with detailed computational definitions provided in Table S2. State occurrence frequencies served as input features for all classification analyses, enabling direct comparison of each method's capacity to generate discriminative representations for cognitive state identification.

### 2.6 EPLSA Application in AD Patients

To evaluate the clinical translational potential of EPLSA in neurodegenerative disorders, we implemented a comprehensive analytical framework using the OASIS‐3 dataset, which included resting‐state fMRI data from individuals at various stages of AD (*n* = 39 scans) and cognitively normal controls (*n* = 233 scans). The analysis pipeline was designed to: (1) identify disease‐specific alterations in brain state organization, (2) quantify changes in dynamic network interactions, and (3) establish relationships between network dynamics and clinical manifestations.

- *Energy Landscape Characterization*: Energy landscapes and corresponding disconnectivity graphs were constructed for both AD and control groups to identify disease‐specific alterations in brain state organization. Local energy minima were extracted to determine stable brain configurations.
- *Brain State Dynamics Analysis*: Three key metrics were computed to characterize pathological alterations in brain dynamics, including state occurrence frequencies, state dwell times, and inter‐state transition probabilities. Between‐group comparisons were conducted using independent‐samples *t*‐tests.
- *Clinical Correlation Assessment*: To establish the clinical relevance of EPLSA‐derived metrics, we examined correlations between brain state dynamics and cognitive performance. Partial Pearson correlation analyses were performed between state‐specific measures (occurrence frequency, dwell time, and transition probabilities) and scores on the Mini‐Mental State Examination (MMSE), Logical Memory Test (LMT), Boston Naming Test (BNT), and the Trail Making Test Part A (TMA) and Part B (TMB), controlling for age and educational level.

### 2.7 Statistical Analysis

This section details all statistical tests and correction procedures used in the study.

- *Between‐group comparisons:* Independent‐sample two‐tailed *t*‐tests were used to compare brain state metrics (state occurrence frequency, dwell time, transition probability) between groups (wakefulness vs. sleep; Alzheimer's disease patients vs. cognitively normal controls).
- *Correlation analysis*: Partial Pearson correlation was used to examine associations between brain state dynamics and cognitive test scores, with age and educational level as nuisance covariates.
- *Classification evaluation:* Random‐forest classifiers with 10‐fold cross‐validation were applied to assess individual‐level classification performance, including accuracy, precision, recall, and F1‐score.
- *Multiple comparison correction:* False Discovery Rate (FDR) correction with *α* = 0.05 was applied for all methodological performance benchmarks (test–retest reliability, task‐specific brain state differentiation, multi‐condition classification). For between‐group comparisons in the Alzheimer's disease analysis, uncorrected *p*‐values are reported.

## 3 Results

### 3.1 Comparative Methodology Evaluation

#### 3.1.1 Task‐Specific Brain State Differentiation

**FIGURE 2:**
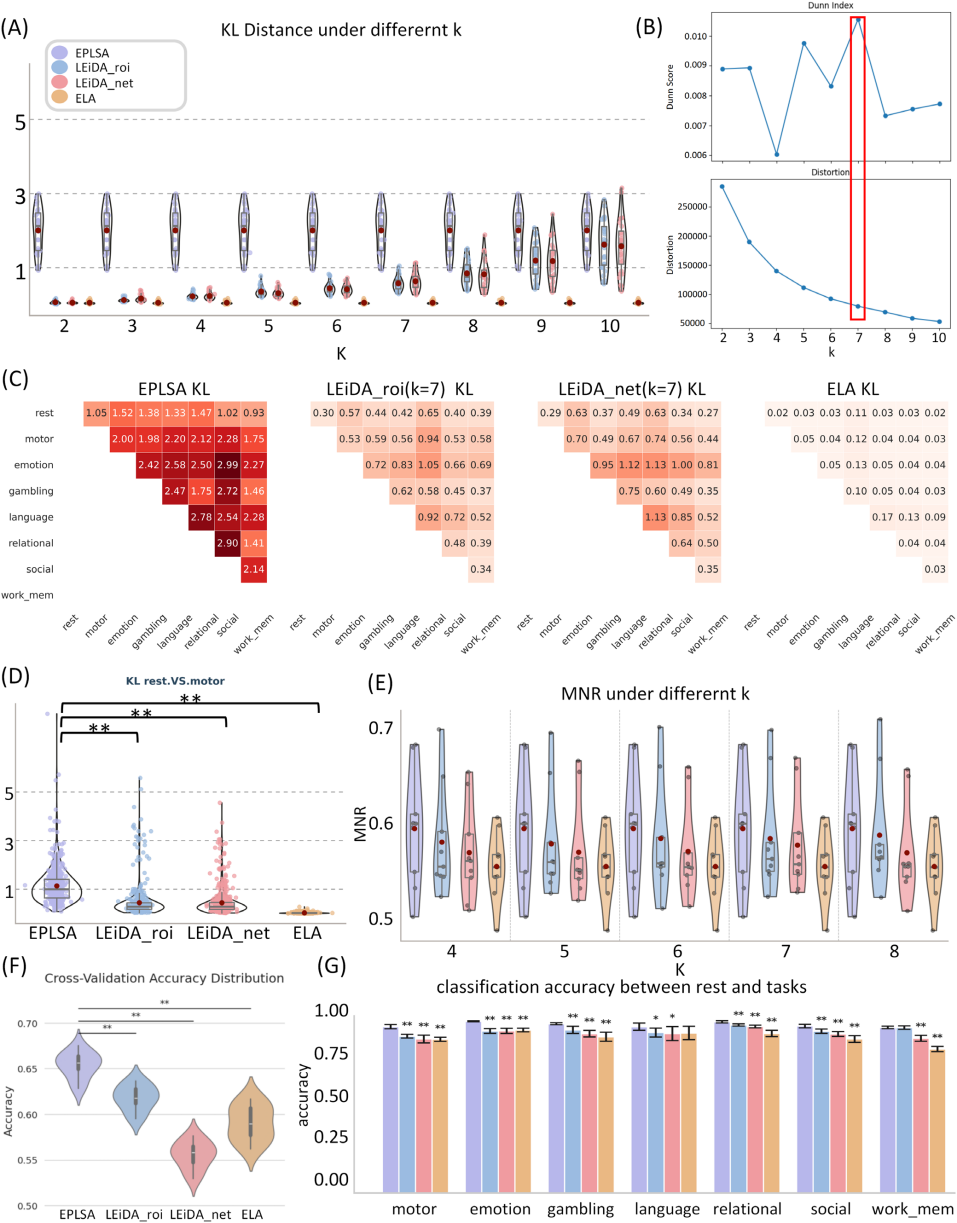
Comparative performance evaluation of brain state estimation methods across multiple analytical dimensions. (A) Symmetrized Kullback–Leibler divergence across varying cluster numbers (*k* = 2–10) for LEiDA‐based approaches. (B) The LEiDA optimal clustering (*k* = 7). (C) Comprehensive KL divergence assessment across all 28 pairwise task comparisons using optimal LEiDA parameterization (*k* = 7); (D) Exemplar comparison of KL divergence between rest and motor task conditions with LEiDA optimal clustering (*k* = 7). (E) Test–retest reliability assessed via discriminability index across varying cluster numbers. (F) Multi‐class classification accuracy for eight HCP conditions (rest plus seven cognitive tasks) using brain state occurrence frequencies as input features. (G) Binary classification accuracy distinguishing rest from cognitive task conditions. All panels share the legend shown in (A). LEiDA‐ROI: Leading Eigenvector Dynamic Analysis applied to individual brain region time series; LEiDA‐NET: Leading Eigenvector Dynamic Analysis applied to network‐averaged time series; ELA: Energy Landscape Analysis based on network‐averaged time series; EPLSA: Energy‐based Phase‐Locking State Analysis (present method). Violin plots illustrate the distribution density and dispersion of the target metric across subjects, with an embedded boxplot (median, quartiles). Error bars in bar plots represent standard deviation (SD) across subjects. Statistical significance is denoted as *p < 0.05, **p < 0.001.

To systematically evaluate the discriminative power of EPLSA relative to established approaches, we computed KL divergence for all pairwise comparisons between brain states across rest and seven cognitive tasks (C_8_^2^ = 28 combinations total). Initial optimization analyses demonstrated that LEiDA performance improved progressively with increasing cluster number *k* (Figure 2A). To determine the optimal k, we quantified clustering quality using two complementary metrics: (1) Dunn Index: A composite metric evaluating clustering validity by balancing intra‐cluster compactness and inter‐cluster separation. A higher Dunn Index indicates that clusters are more tightly clustered (small intra‐cluster distance) and more distinct from each other (large inter‐cluster distance), reflecting better clustering quality. (2) Distortion: A measure of within‐cluster variance. A lower Distortion value indicates that samples within the same cluster are more concentrated, representing more homogeneous clustering results. Traversing candidate *k* values (2–10), we found convergent evidence that *k* = 7 yielded the global maximum Dunn Index and local minimum Distortion (Figure 2B), identifying *k* = 7 as the optimal configuration. When employing this optimal clustering parameter, EPLSA exhibited significantly higher KL divergence values across all 28 comparison cases relative to benchmark methods (Figure 2C), with this superiority maintained consistently across alternative *k* values (Figure S3). Exemplifying this enhanced discrimination capability, the rest‐versus‐motor task comparison revealed significantly superior task differentiation for EPLSA compared to all alternative methods (*p* < 0.001, FDR‐corrected, Figure 2D). This pattern of superior discriminability was replicated across the remaining 27 task comparisons, with EPLSA consistently demonstrating significantly enhanced state distinguishability relative to LEiDA variants and traditional ELA approaches (Figure S4). Importantly, these findings were robustly replicated across both phase‐encoding directions, with left‐to‐right (LR) and right‐to‐left (RL) acquisition protocols yielding identical patterns of methodological superiority (Figure S5).

#### 3.1.2 Individual‐Level Classification Performance

Comprehensive evaluation of predictive capability employed occurrence frequency metrics as feature variables for classification of eight distinct conditions (rest plus seven task). EPLSA demonstrated superior performance across all evaluated metrics (*p* < 0.001, FDR‐corrected), including prediction accuracy (Figure 2F), recall, precision, and F1‐score (Figure S8A). Particularly noteworthy was EPLSA's enhanced classification accuracy for rest‐versus‐task distinctions (Figure 2G), indicating exceptional sensitivity for detecting subtle differences between baseline and task‐engaged brain states. This discriminative capacity extended to resting‐state prediction, where EPLSA achieved superior accuracy compared to all benchmark approaches (Figure S8B). These findings possess particular clinical significance given the fundamental importance of resting‐state fMRI characterization for understanding baseline neural activity patterns and their alterations in neurological and psychiatric conditions.

#### 3.1.3 Test–Retest Reliability Assessment

The assessment of test–retest reliability through discriminability analysis revealed that EPLSA maintained high measurement consistency comparable to state‐of‐the‐art LEiDA variants (Figure 2E and Figure S6, *p* > 0.05, FDR‐corrected). These findings indicate that our framework successfully retains the robust reproducibility of phase‐based dynamics while providing the added value of significantly enhanced state differentiation and predictive power across cognitive and clinical conditions. Correlation analyses further demonstrated a significant positive relationship between time series duration and test–retest reliability across all methodological approaches (Figure S7 and Table S3). Notably, language task conditions exhibited deviation from this general trend, potentially attributable to the heightened pattern complexity inherent in linguistic processing demands (Capouskova et al. 2022). These reliability findings underscore EPLSA's capacity to generate consistent brain state characterizations across repeated measurements, a critical requirement for longitudinal and clinical applications.

### 3.2 Discrimination Between Sleep–Wake Conditions

**FIGURE 3:**
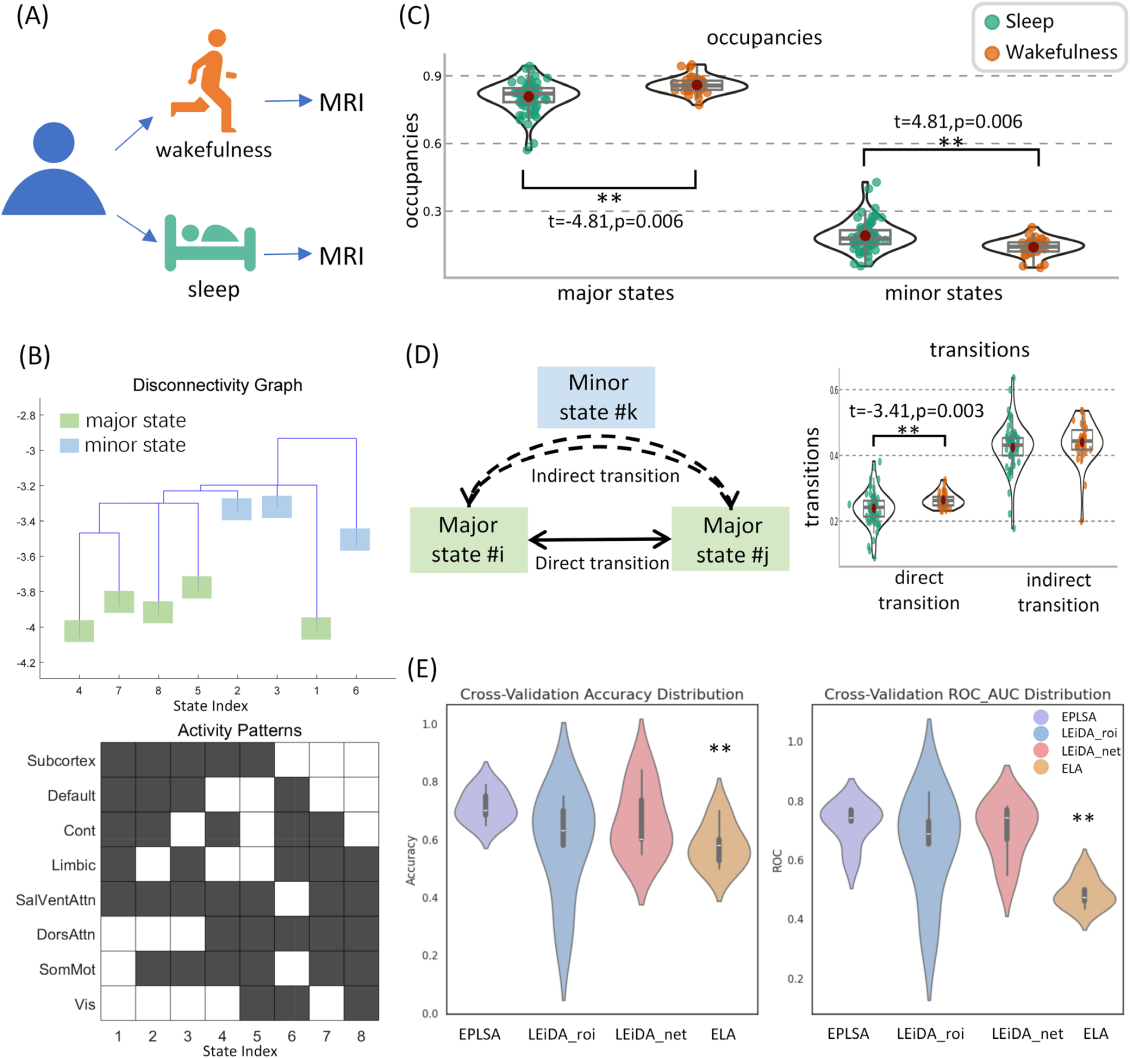
EPLSA characterization of sleep–wake state transitions and brain dynamics. (A) Experimental paradigm distinguishing sleep and wakefulness conditions in the Natural Sleep dataset. (B) Energy landscape disconnectivity graphs with corresponding activity patterns at local energy minima, illustrating state‐specific brain configurations. Note: In activity pattern plots, black squares represent inactive network states (−1), and white squares represent active network states (+1). (C) Differential occurrence frequencies of major versus minor brain states between sleep and wake conditions, demonstrating significant redistribution toward higher‐energy configurations during sleep. (D) Transition pathway analysis distinguishing direct inter‐state transitions from indirect pathways mediated by minor states, revealing significantly reduced direct transition probabilities during sleep while maintaining comparable indirect transition rates; (E) Machine learning classification performance metrics (accuracy and area under ROC curve) for sleep–wake state discrimination using random forest algorithms with EPLSA‐derived features. Violin plots illustrate the distribution density and dispersion of the target metric across subjects, with an embedded boxplot (median, quartiles). Error bars in bar plots represent standard deviation (SD) across subjects. Statistical significance is denoted as *p < 0.05, **p < 0.001.

To further establish the generalizability and validity of EPLSA, we applied this approach to the Natural Sleep Dataset containing simultaneous recordings of wakefulness and sleep states. The analytical framework identified eight distinct brain states, which were systematically categorized into primary and secondary configurations based on basin size distributions—defined as the fractional proportion of brain activity patterns belonging to each attractor basin (Figure 3B). Sleep‐related alterations in brain state dynamics revealed significant reductions in primary state occurrence frequency during sleep relative to wakefulness (*t* = −4.81, *p* = 0.006, FDR‐corrected), accompanied by corresponding increases in secondary state prevalence (*t* = 4.81, *p* = 0.006, FDR‐corrected) (Figure 3C). Analysis of state transition dynamics demonstrated significantly decreased direct transition probabilities during sleep (*t* = −3.41, *p* = 0.003, FDR‐corrected) (Figure 3D).

For the sleep–wake classification analysis, the optimal cluster number k for the LEiDA benchmarks was determined independently on the Natural Sleep dataset using the Dunn Index and Distortion. Comparative performance evaluation demonstrated EPLSA's superior and more stable discriminative capability for sleep–wake classification relative to traditional methodological approaches (Figure 3E). Individual brain state comparisons revealed significantly reduced dwell times for states associated with subcortical regions and default mode network during sleep conditions (Figure S9). These findings collectively demonstrate EPLSA's enhanced sensitivity to physiologically meaningful alterations in brain state dynamics across consciousness levels.

### 3.3 Brain States Characterization in AD Patients

**FIGURE 4:**
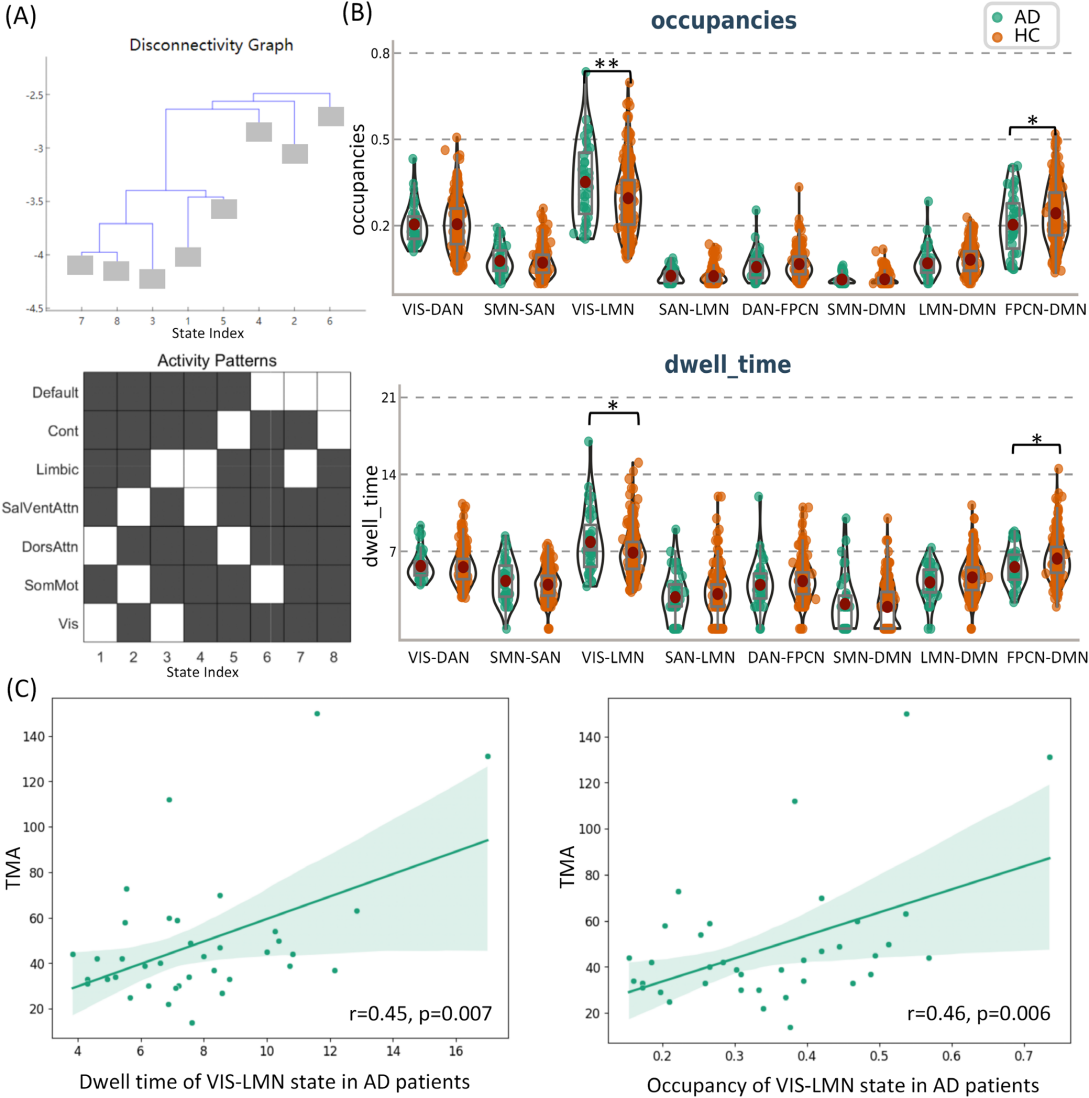
EPLSA reveals altered brain state dynamics and clinical correlations in Alzheimer's disease. (A) Energy landscape disconnectivity graphs for the OASIS‐3 dataset with activity patterns corresponding to local energy minima, highlighting disease‐specific alterations in brain state organization. In activity pattern plots, black squares represent inactive network states (−1), and white squares represent active network states (+1). (B) Between‐group comparisons of brain state occurrence frequencies and dwell times in AD patients versus healthy controls, demonstrating differential occupancy patterns across functional configurations. (C) Clinical correlation analysis examining relationships between EPLSA‐derived brain state metrics and Trail Making Test PART A (TMA) scores in AD patients, revealing significant associations between network dynamics and cognitive performance. Violin plots illustrate the distribution density and dispersion of the target metric across subjects, with an embedded boxplot (median, quartiles). Error bars in bar plots represent standard deviation (SD) across subjects. Statistical significance is denoted as *p < 0.05, **p < 0.001. DAN = Dorsal Attention Network; DMN = Default Mode Network; FPCN = Frontoparietal Control Network; LMN = Limbic Network; SAN = Salience Network; SMN = Sensorimotor Network; VIS = Visual Network.

Following comprehensive validation of EPLSA's methodological advantages across multiple evaluation dimensions, we employed this framework to systematically characterize whole‐brain dynamic patterns in AD patients relative to cognitively normal adults. The analytical framework identified eight distinct metastable brain states, each characterized by unique network activation patterns (Figure 4A). State were named based on their dominant active network configurations; for example, the state showing simultaneous activation of the visual network (VIS) and dorsal attention network (DAN) was designated the “VIS‐DAN state,” with similar conventions applied to all identified states.

Group comparisons revealed significant alterations in both the frequency and temporal duration of specific brain states in AD patients. The VIS‐LMN state demonstrated significantly higher occurrence frequency (*p* = 0.007, uncorrected) and longer duration (*p* = 0.047, uncorrected) in AD patients compared to cognitively normal controls, potentially reflecting the reduced energy barriers associated with this particular configuration. In contrast, FPCN‐DMN states exhibited significantly lower occurrence frequencies (*p* = 0.025, uncorrected) and reduced dwell times (*p* = 0.020, uncorrected) in AD patients (Figure 4B).

Correlation analyses between brain state dynamics and cognitive performance identified significant associations between VIS‐LMN state metrics and Trail Making Test Part A (TMA) scores in AD patients. Specifically, longer dwell time and higher occupancy in the VIS‐LMN state were positively correlated with TMA scores (*r* = 0.45, *p* = 0.007; *r* = 0.46, *p* = 0.006, respectively) (Figure 4C). These findings indicate that the increased occurrence frequency and extended duration of the VIS‐LMN state are associated with more severe cognitive impairment in AD patients, as higher TMA scores reflect poorer cognitive performance.

## 4 Discussion

The investigation of spatiotemporal dynamics in the human brain represents a fundamental endeavor in contemporary neuroscience, with profound implications for elucidating pathophysiological mechanisms and developing targeted neuroregulatory interventions. The establishment of robust analytical frameworks for characterizing brain dynamics is therefore essential for advancing our understanding of neurological disorder progression and phenotypic manifestations. In this study, we introduce Energy‐based Phase‐Locking State Analysis (EPLSA), a novel computational framework that synergistically integrates phase‐coupling dynamics with energy landscape theory to comprehensively characterize brain dynamics. By constructing an energy landscape on the leading eigenvector of phase coherence matrices, EPLSA effectively captures spatiotemporal interactions while circumventing the inherent dimensionality constraints associated with conventional dynamic functional connectivity approaches. Our empirical findings demonstrate that EPLSA exhibits superior performance relative to established methodologies across multiple evaluation metrics, including task differentiation, test–retest reliability, and predictive accuracy, while simultaneously revealing distinctive brain state dynamics associated with sleep–wake transitions and Alzheimer's disease pathology. Critically, the EPLSA‐derived features serve as sensitive biomarkers for consciousness states and neurological disorders, demonstrating superior discriminative power compared to conventional methods and offering clinically translatable indicators for early detection and differential diagnosis of neuropathological conditions.

The methodological distinctions between LEiDA and ELA reflect their respective theoretical foundations and complementary capabilities in characterizing different dimensions of brain dynamics. ELA, grounded in statistical physics principles, quantifies multi‐stability by conceptualizing brain states as attractors within an energy landscape framework, thereby facilitating direct computation of transition probabilities and state stability metrics (Ezaki et al. 2017; Watanabe, Hirose, et al. 2014; Watanabe, Masuda, et al. 2014). However, conventional ELA implementations typically employ binarized amplitude‐based activity patterns, potentially overlooking critical phase‐coupling dynamics that mediate large‐scale neural coordination (Cabral et al. 2017). Conversely, LEiDA utilizes phase coherence measures to identify transient synchronization states, rendering it particularly sensitive to rapid functional network reconfigurations (Deco et al. 2019; Lord et al. 2019). Nevertheless, LEiDA lacks a formal theoretical framework for quantifying state stability or the energy barriers underlying state transitions, limiting its utility for understanding the thermodynamic constraints governing brain dynamics.

To bridge these methodological gaps, we propose EPLSA as a unifying framework that addresses these fundamental limitations by constructing energy landscapes derived from phase‐locking patterns. EPLSA incorporates phase‐coupling dynamics while preserving critical information about inter‐regional synchronization (Cabral et al. 2017; Vidaurre et al. 2018) and extends LEiDA by introducing formal energy metrics derived from pairwise maximum entropy model. The brain states identified by EPLSA, characterized as local minima in the energy landscape, align conceptually with “ghost attractors”—weakly stable, transient configurations that recurrently emerge in spontaneous brain activity (Vohryzek et al. 2020). By projecting high‐dimensional phase‐locking matrices into low‐dimensional energy landscapes, the method establishes direct mappings between dynamical system theory and neural phenomena: (1) these ghost attractor basins correspond to functionally coherent network ensembles, (2) potential well depth quantifies state metastability through its relationship to energy barriers, and (3) saddle points represent critical transition states between functional configurations. EPLSA thus provides a unified framework that retains LEiDA's sensitivity to dynamic coupling and incorporates the thermodynamic form of ELA, providing quantitative tools for assessing state stability and transition difficulty through energy metrics. This integration captures the fundamental trade‐off between functional segregation and integration in whole‐brain dynamics, offering unprecedented insights into the energetic constraints governing neural network organization.

Dynamic analysis of brain states during sleep revealed a significant increase in the occurrence frequency of minor states and a notable decrease in direct state transition probabilities, suggesting enhanced brain dynamics characterized by frequent crossings of energy barriers and heightened instability during sleep. This indicates a system exploring a broader landscape of weakly stable ghost attractors, with reduced dwell time in the most stable basins. Specifically, the reduced dwell time in stable, low‐energy basins and the increased exploration of high‐energy configurations indicate a departure from stereotyped neural pathways. The decreased probability of direct major‐to‐major state transitions does not imply fewer overall state changes; rather, it reflects a diversification of transition paths, as the system more frequently traverses minor, intermediate states instead of taking direct routes between major attractors. Previous investigations have documented substantial state transitions during sleep (Guo et al. 2022; Horovitz et al. 2008, 2009), and this increased instability may reflect the brain's exploration of broader functional configurations, potentially facilitating memory consolidation and neural recovery. Furthermore, previous research has demonstrated that transitions from sleep to wakefulness are more readily facilitated than those from wakefulness to sleep (Deco et al. 2019), a finding consistent with our results indicating a higher prevalence of high‐energy states during sleep and a more natural transition from high‐ to low‐energy states. Additionally, we observed significant reductions in the dwell times of brain states associated with the default mode network (DMN) during sleep, which aligns with reported declines in DMN connectivity during sleep states (De Havas et al. 2012; Sämann et al. 2010). These findings collectively provide novel insights into the neural mechanisms underlying sleep and its functional significance, suggesting that sleep represents a period of enhanced exploration of the brain's functional repertoire.

Analysis of brain dynamics in patients with AD relative to healthy controls revealed significant reductions in both the occurrence frequency and dwell time of brain states associated with the DMN and the FPCN. These results indicate marked declines in DMN and FPCN network activity in AD patients, consistent with established literature documenting these networks' vulnerability in neurodegenerative processes (Zhao et al. 2019; Perovnik et al. 2022). By contrast, the same comparative analysis uncovered a complementary pattern in other functional networks: AD patients exhibited significant elevations in both the occurrence frequency and dwell time of brain states linked to the visual network (VIS) and the limbic network (LMN). The VIS‐LMN state has the lowest energy. By unifying phase‐synchrony and energy landscape principles, EPLSA reveals that AD pathology is characterized by a thermodynamic shift toward low‐energy sensory “traps” and a constricted functional repertoire driven by underlying synaptic asynchrony (Huang et al. 2021; Singh et al. 2023; Tessadori et al. 2025). Furthermore, the frequency of the brain states exhibited significant positive correlations with cognitive performance, suggesting their potential utility as predictors of cognitive impairment severity in AD patients, a finding consistent with prior research establishing links between network dysfunction and AD symptoms (Huang et al. 2021). These observations underscore the potential of altered VIS activity as a robust pathological marker for AD, providing quantitative metrics that may facilitate early detection and disease monitoring (Huang et al. 2021; Singh et al. 2023; Varangis et al. 2025). Notably, such dynamical alterations in state occupancy and dwell time are not unique to AD, but rather align with core findings from other neuropsychiatric disorders investigated via phase‐based dynamical analysis frameworks. For example, Farinha et al. (2022) applied LEiDA to schizophrenia and reported altered fractional occupancy, dwell times, and transition probabilities, demonstrating the broader applicability of dynamical systems approaches to clinical populations. The present EPLSA framework builds on and extends these phase‐based analyses by not only capturing these key temporal dynamic metrics but also quantifying the underlying energy landscape, thereby mechanistically linking observed dynamic alterations (e.g., the increased prevalence of the low‐energy VIS‐LMN state in AD) to fundamental changes in the thermodynamic constraints governing the brain's state space transitions. The energy landscape perspective offered by EPLSA reveals that AD pathology fundamentally alters the thermodynamic constraints governing brain state transitions, with implications for understanding disease progression and potential therapeutic targets.

While we have demonstrated the advantages and practical utility of EPLSA through multiple validation approaches, several limitations and considerations warrant careful attention. First, our methodology is fundamentally grounded in energy landscape theory, which inherently constrains the optimal number of variables for analysis to a range of 6–15, depending on the length of the time series data. When applied to datasets with a larger number of variables, the computational cost increases significantly, and the interpretability of results becomes more challenging (Ezaki et al. 2017). Second, although our study utilized a relatively robust sample size of 590 participants from the Human Connectome Project (HCP) dataset, recent research suggests that studies aiming to reliably capture brain‐behavior relationships should target sample sizes in the tens of thousands (Marek et al. 2022). Third, the retention of only the leading eigenvector necessarily results in information loss, potentially overlooking secondary modes of synchronization that may contribute to brain state characterization. Lastly, we did not perform supplementary analyses without GSR in the current study, and thus cannot fully rule out the potential impact of GSR on the detection of global phase synchronization states and the magnitude of group‐level differences in brain dynamics. Future studies should systematically investigate the effect of GSR and other preprocessing choices on EPLSA‐derived brain state metrics, including direct comparisons of results with and without GSR across diverse datasets. Finally, further validation using additional clinical or longitudinal datasets is necessary to strengthen the generalizability and reliability of our findings across diverse populations and clinical conditions.

In summary, this study introduces EPLSA as a unified framework that bridges phase‐coupling dynamic and thermodynamic stability principles to characterize brain states with high precision and theoretical grounding. By constructing an energy landscape from BOLD phase synchrony, EPLSA achieves three key advances: (1) superior state discriminability with improved task‐decoding accuracy compared to conventional methods; (2) quantifiable stability metrics that offer novel neurodynamic insights into the energetic constraints governing brain function; (3) strong clinical translatability, as evidenced by excellent performance in sleep–wakefulness classification and Alzheimer's disease characterization. These results establish that phase‐derived energy barriers fundamentally govern cognitive state transitions, providing a new theoretical framework for understanding brain dynamics across health and disease. Future research should explore the translational potential of this approach across diverse demographic groups and endeavor to uncover related biomarkers for diagnosis and monitoring of neurological disorders, ultimately contributing to the development of precision medicine approaches in neurology and psychiatry.

## Author Contributions

C.Y. and T.M. conceptualized this work. Z.D., C.Y. and S.H. developed the analytical methods, analyzed the data, prepared the figures, and drafted the manuscript. C.R., T.G. and S.C. collected and co‐analyzed the data.

## Funding

This study is supported by grants from the Guangxi Science and Technology Base and Special Talent Program (GuikeAD23026245), National Natural Science Foundation of P.R. China (62106113, 62276081, 62466006), Basic and Applied Basic Research Foundation of Guangdong Province (2023A1515010792, 2023B1515120065), Shenzhen Science and Technology Program (GXWD20231129121139001, JCYJ20240813110522029, CJGJZD20230724093959002).

## Ethics Statement

This study was conducted in strict accordance with the Declaration of Helsinki and international ethical principles for human subjects research. All analyses were performed exclusively on publicly available de‐identified neuroimaging datasets, and no additional human subjects experiments were conducted. The Human Connectome Project (HCP) S1200 dataset, Natural Sleep dataset, and OASIS‐3 dataset were all collected with approval from their respective institutional review boards. Written informed consent was obtained from all participants or their legal guardians, and strict anonymization protocols were implemented to protect participant privacy.

## Supporting information


**Figure S1:** The methodological description of the Leading Eigenvector Dynamic Analysis (LEiDA). (A) We use the LEiDA method, where for every time point, *t*, in every brain region of each participant we extract the BOLD signal, compute the phase of the BOLD signal. (B) Compute the BOLD phase coherence matrix, PC(*t*), between brain regions, and extract the eigenvectors V1(*t*) of this matrix. (C) We take the leading eigenvector V1(*t*) as a low‐dimensional representation of the BOLD phase‐locking patterns over time. (D) To identify recurrent phase‐locking patterns, we apply a clustering algorithm (*k*‐means) to divide the sample into a predefined number of clusters k (here, *k* = 4). Each cluster is represented by a central vector (yellow, green, blue, black), which we take to represent a recurrent pattern of phase coherence, or brain state. The color map of the glassbrain indicates the strength of functional connectivity (FC) between each pair of brain regions.
**Figure S2:** The methodological description of the Energy Landscape Analysis (ELA). This figure was modified from Figure 1 of ref. (Ezaki et al. 2017). (A) Classified ROIs into seven functionally different brain systems. (B) Calculated their average network activity. (C) The fMRI signal at each network and each time point is binarized into 1 (active) or −1 (inactive). (D) The pairwise MEM model (i.e., Boltzmann distribution) is fitted to the empirical distribution of the $2^{N}$ activity patterns. The energy value is also obtained for each activity pattern. (E) Relationships between activity patterns that are energy local minimums are summarized into a disconnectivity graph. (F) Schematic of the energy landscape. Each local minimum corresponds to the bottom of a basin. The borders between attractive basins of different local minimums are shown by the dotted curves. Any activity pattern belongs to the basin of a local minimum. Brain dynamics can be interpreted as the motion of a “ball” constrained on the energy landscape.
**Figure S3:** The comparison of KL divergence of different methods when *k* = 2–10 (except *k* = 7) is selected by the LEiDA method based on the LR group scan results.
**Figure S4:** The comparison of KL divergence of different methods and tasks when *k* = 7 is selected by the LEiDA method based on the LR group scan results.
**Figure S5:** The comparison of KL divergence of different methods when *k* = 2–10 is selected by the LEiDA method based on the RL group scan results.
**Figure S6:** MNR comparison of different methods for *k* (2–10).
**Figure S7:** MNR comparison of different methods for each task.
**Figure S8:** Prediction performance except classification accuracy of rest from other tasks and classification accuracy in different tasks (*p < 0.05(FDR); **p < 0.001(FDR)).
**Figure S9:** Between‐group comparisons of brain state dwell times in sleep versus wakefulness.
**Table S1:** Amyloid Positivity Thresholds by PET Tracer (see Su et al. 2019).
**Table S2:** Definitions for different measurements.
**Table S3:** The length of timeseries and MNR value in methods of rest and seven tasks.

## Data Availability

The data that support the findings of this study are available in Human Connectome Project at http://www.humanconnectomeproject.org/. These data were derived from the following resources available in the public domain: Human Connectome Project.
