# Supplementary material for: Energy‐Based Phase‐Locking State Analysis in Brain State Identification
Source: Hum Brain Mapp. 2026 Jun 5;47(8):e70558. doi: 10.1002/hbm.70558 (PMC13238561; doi:10.1002/hbm.70558)
Supplement: Supplementary file 1 — Figure S1: The methodological description of the Leading Eigenvector Dynamic Analysis (LEiDA). (A) We use the LEiDA method, where for every time point, t, in every brain region of each participant we extract the BOLD signal, compute the phase of the BOLD signal. (B) Compute the BOLD phase coherence matrix, PC(t), between brain regions, and extract the eigenvectors V1(t) of this matrix. (C) We take the leading eigenvector V1(t) as a low‐dimensional representation of the BOLD phase‐locking patterns over time. (D) To identify recurrent phase‐locking patterns, we apply a clustering algorithm (k‐means) to divide the sample into a predefined number of clusters k (here, k = 4). Each cluster is represented by a central vector (yellow, green, blue, black), which we take to represent a recurrent pattern of phase coherence, or brain state. The color map of the glassbrain indicates the strength of functional connectivity (FC) between each pair of brain regions. Figure S2: The methodological description of the Energy Landscape Analysis (ELA). This figure was modified from Figure 1 of ref. (Ezaki et al. 2017). (A) Classified ROIs into seven functionally different brain systems. (B) Calculated their average network activity. (C) The fMRI signal at each network and each time point is binarized into 1 (active) or −1 (inactive). (D) The pairwise MEM model (i.e., Boltzmann distribution) is fitted to the empirical distribution of the 2N activity patterns. The energy value is also obtained for each activity pattern. (E) Relationships between activity patterns that are energy local minimums are summarized into a disconnectivity graph. (F) Schematic of the energy landscape. Each local minimum corresponds to the bottom of a basin. The borders between attractive basins of different local minimums are shown by the dotted curves. Any activity pattern belongs to the basin of a local minimum. Brain dynamics can be interpreted as the motion of a “ball” constrained on the energy landscape. Figure [file HBM-47-e70558-s001.docx]

**Supplementary Files**

**Participants inclusion criteria for OASIS-3 dataset.**

OASIS-3 is a compilation of MRI and PET imaging and related clinical data for 1098 participants who were collected across several ongoing studies in the Washington University Knight Alzheimer Disease Research Center over the course of 15 years. Participants include 605 cognitively normal adults and 493 individuals at various stages of cognitive decline ranging in age from 42 to 95 years (LaMontagne et al., 2019).

All participants included in the analysis first meet OASIS-3’s basic requirements: (1) Complete at least one Uniform Data Set (UDS)-compliant clinical assessment, with baseline imaging (MRI/PET) and clinical evaluation interval ≤ 6 months; (2) Pass imaging quality control (PET via PET Unified Pipeline [PUP]; MRI via FreeSurfer segmentation, labeled "pass" or "pass with edits"); (3) Comply with anonymization protocols and provide IRB-approved informed consent.

- **Healthy Control (HC) Group:**

Cognitive status: Baseline and 12-month follow-up Clinical Dementia Rating (CDR) = 0; Mini-Mental State Examination (MMSE) ≥27, Logical Memory Test ≥15/25 (normal for age) (LaMontagne et al., 2019).

Amyloid level: Defined by tracer-specific Centiloid (CL) thresholds (amyloid-negative): For [¹¹C]-Pittsburgh Compound B (PIB), CL < 16.4; for [¹⁸F]-Florbetapir (AV45), CL < 20.6. Specific thresholds corresponding to these CL values (by tracer and correction status) are shown in Supplementary Table S1.

- **AD-related Cognitive Impairment Group:**

Cognitive status & diagnosis: Baseline CDR ≥0.5 (very mild) or 1 (mild); clinical diagnosis of "AD dementia" or "MCI due to AD" (exclude non-AD etiologies). CDR=0.5 participants need cognitive progression (e.g., MMSE decline ≥3) within 6–12 months (LaMontagne et al., 2019).

Amyloid level: Defined by tracer-specific Centiloid (CL) thresholds (amyloid-positive): For [¹¹C]-Pittsburgh Compound B (PIB), CL ≥ 16.4; for [¹⁸F]-Florbetapir (AV45), CL ≥ 20.6. Specific thresholds corresponding to these CL values are shown in Supplementary Table S1.

We found no significant age difference between HC and AD groups.

**Figure S1.** The methodological description of the Leading Eigenvector Dynamic Analysis (LEiDA). (A) We use the LEiDA method, where for every time point, t, in every brain region of each participant we extract the BOLD signal, compute the phase of the BOLD signal. (B) Compute the BOLD phase coherence matrix, PC(t), between brain regions, and extract the eigenvectors V1(t) of this matrix. (C) We take the leading eigenvector V1(t) as a low-dimensional representation of the BOLD phase-locking patterns over time. (D) To identify recurrent phase-locking patterns, we apply a clustering algorithm (k-means) to divide the sample into a predefined number of clusters k (here, k = 4). Each cluster is represented by a central vector (yellow, green, blue, black), which we take to represent a recurrent pattern of phase coherence, or brain state. The color map of the glassbrain indicates the strength of functional connectivity (FC) between each pair of brain regions.

***
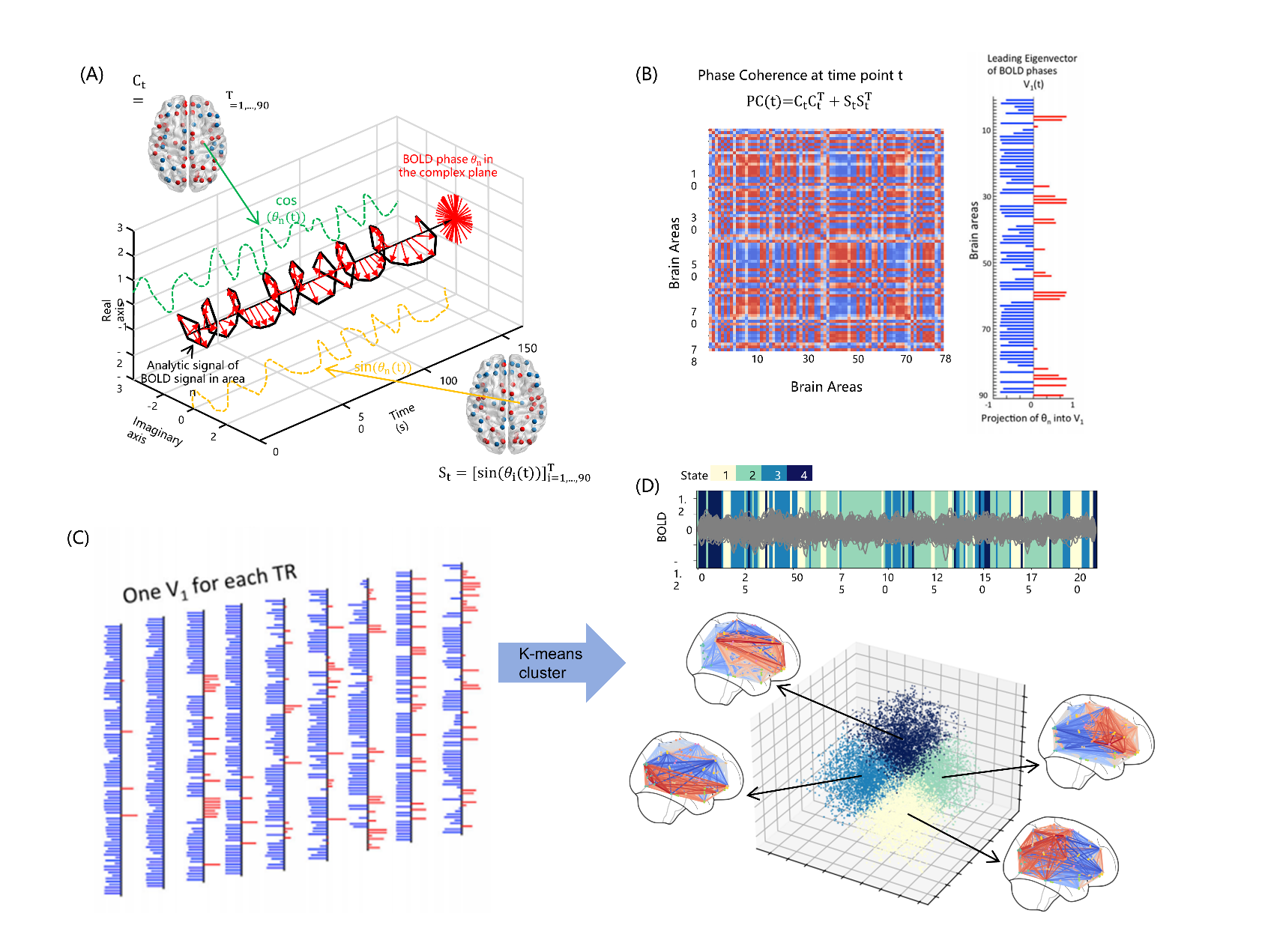
***

**Figure S2.** The methodological description of the Energy Landscape Analysis (ELA). This figure was modified from Figure 1 of ref (Ezaki et al., 2017). (A) Classified ROIs into seven functionally different brain systems. (B) Calculated their average network activity. (C) The fMRI signal at each network and each time point is binarized into 1 (active) or −1 (inactive). (D) The pairwise MEM model (i.e. Boltzmann distribution) is fitted to the empirical distribution of the $2^{N}$ activity patterns. The energy value is also obtained for each activity pattern. (E) Relationships between activity patterns that are energy local minimums are summarized into a disconnectivity graph. (F) Schematic of the energy landscape. Each local minimum corresponds to the bottom of a basin. The borders between attractive basins of different local minimums are shown by the dotted curves. Any activity pattern belongs to the basin of a local minimum. Brain dynamics can be interpreted as the motion of a ‘ball’ constrained on the energy landscape.

***
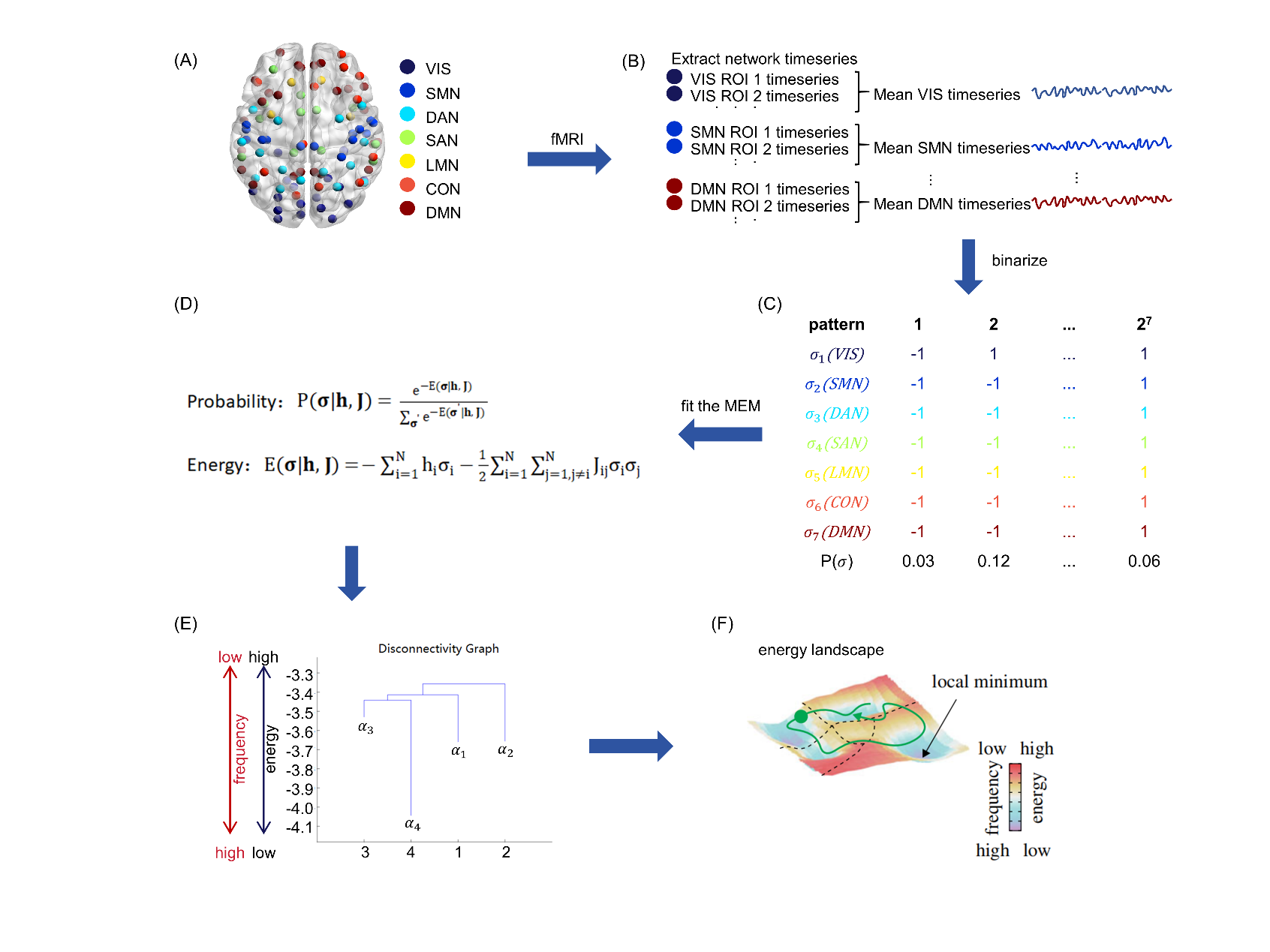
***

**Figure S3.** The comparison of KL divergence of different methods when k = 2 - 10 (except k = 7) is selected by the LEiDA method based on the LR group scan results.


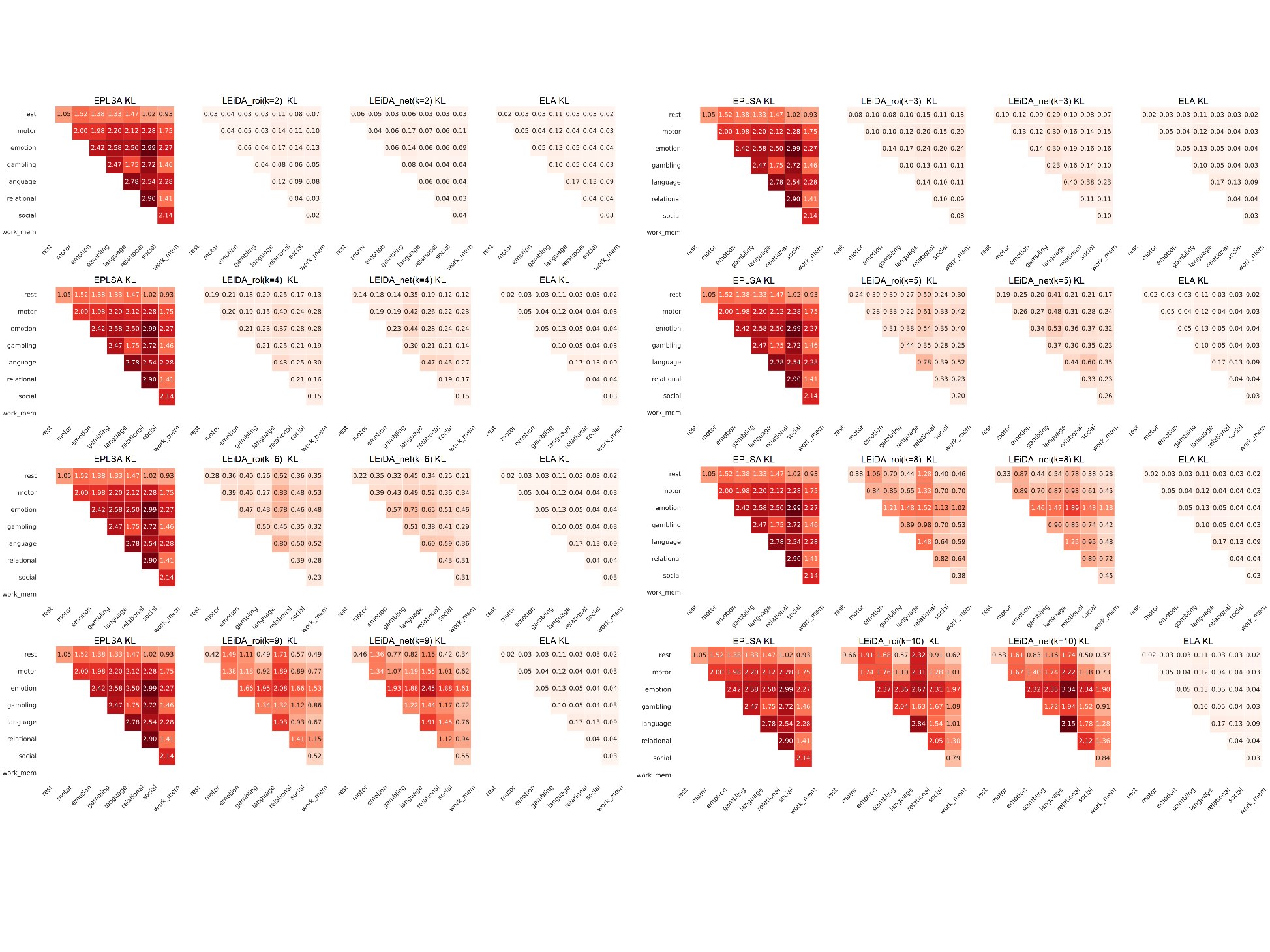


**Figure S4.** The comparison of KL divergence of different methods and tasks when k = 7 is selected by the LEiDA method based on the LR group scan results.


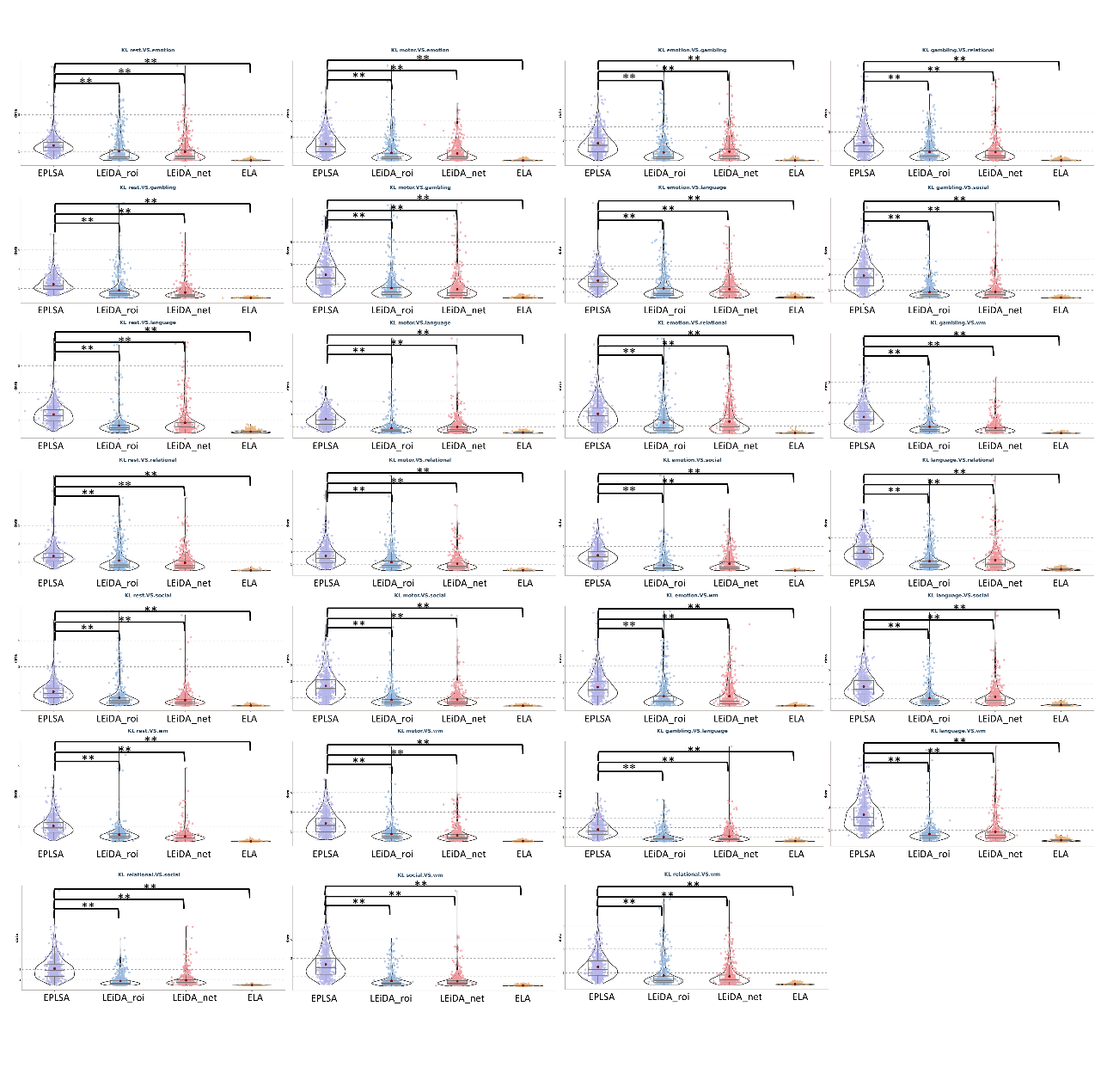


**Figure S5.** The comparison of KL divergence of different methods when k = 2 - 10 is selected by the LEiDA method based on the RL group scan results.


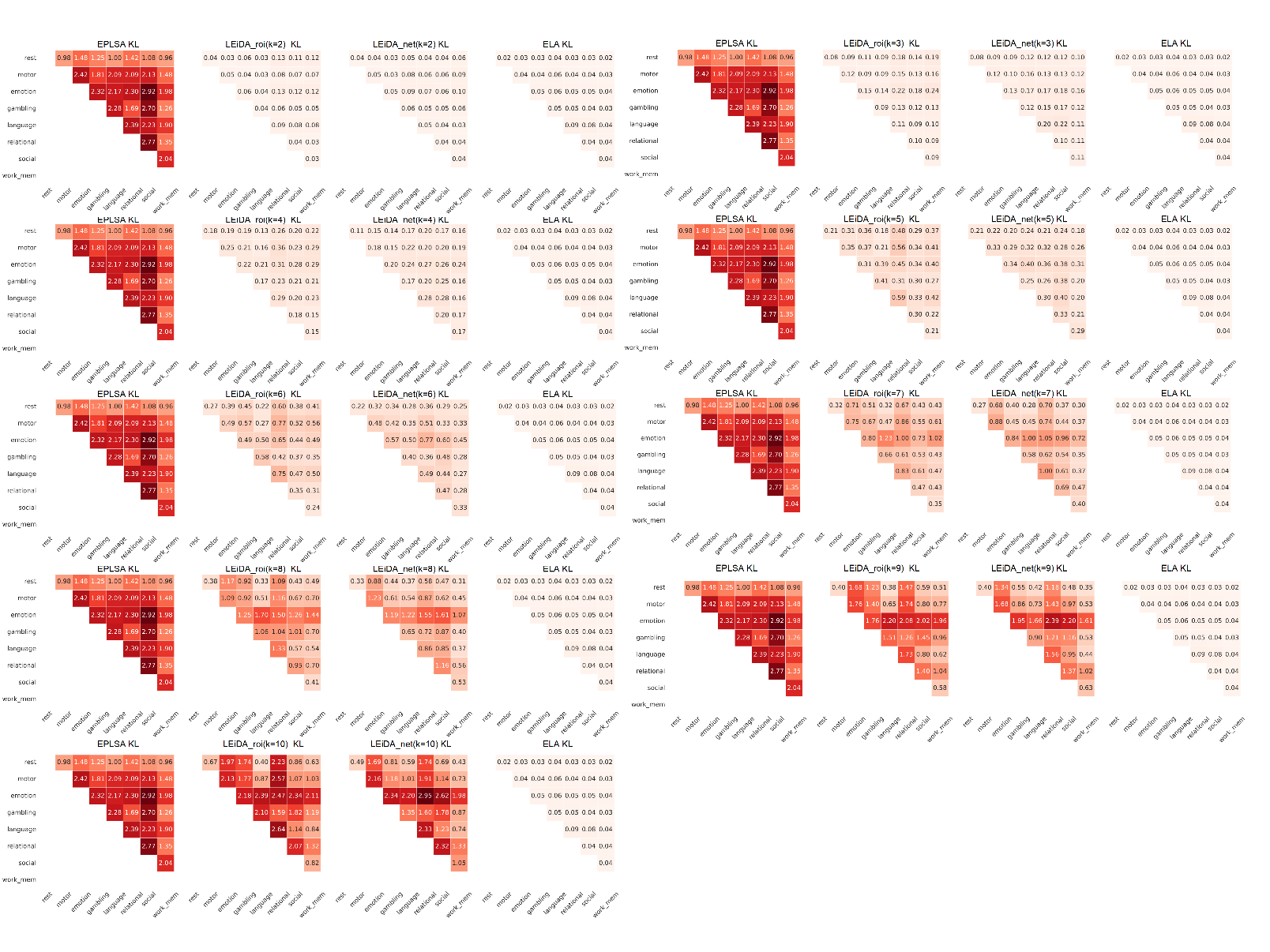


**Figure S6.** MNR comparison of different methods for k (2-10).


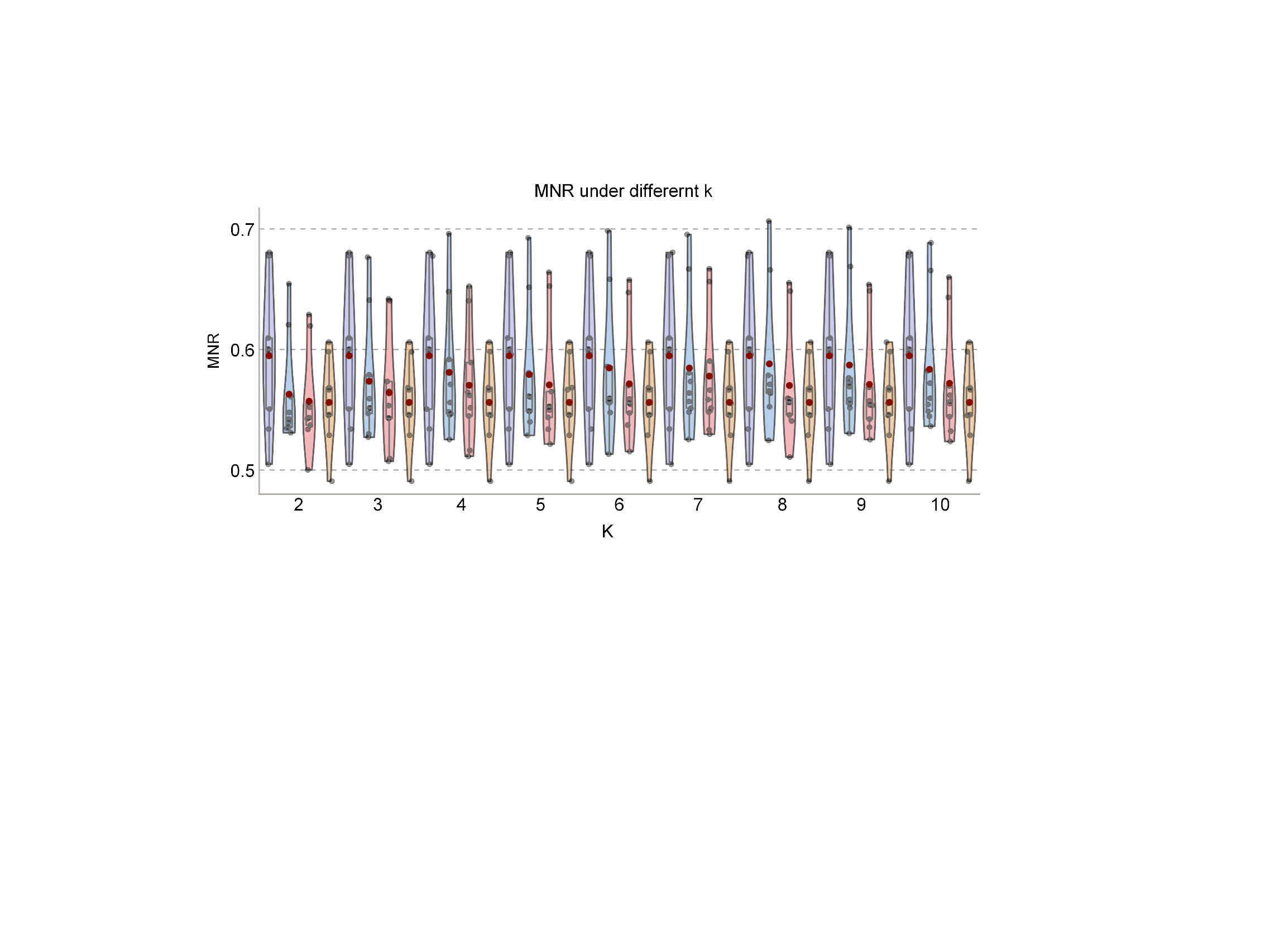


**Figure S7.** MNR comparison of different methods for each task.


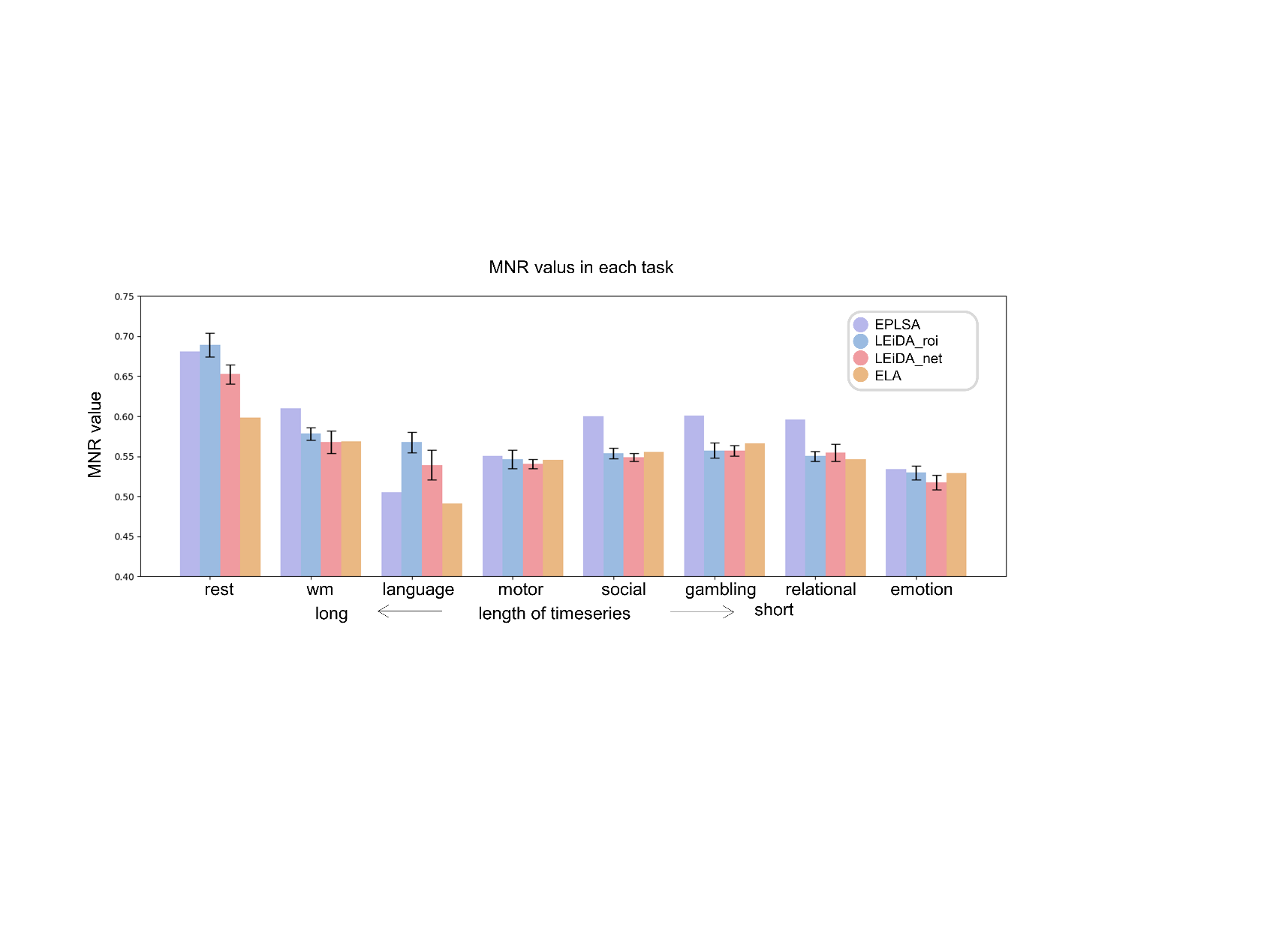


**Figure S8.** Prediction performance except classification accuracy of rest from other tasks and classification accuracy in different tasks. (*p < 0.05(FDR); **p < 0.001(FDR))


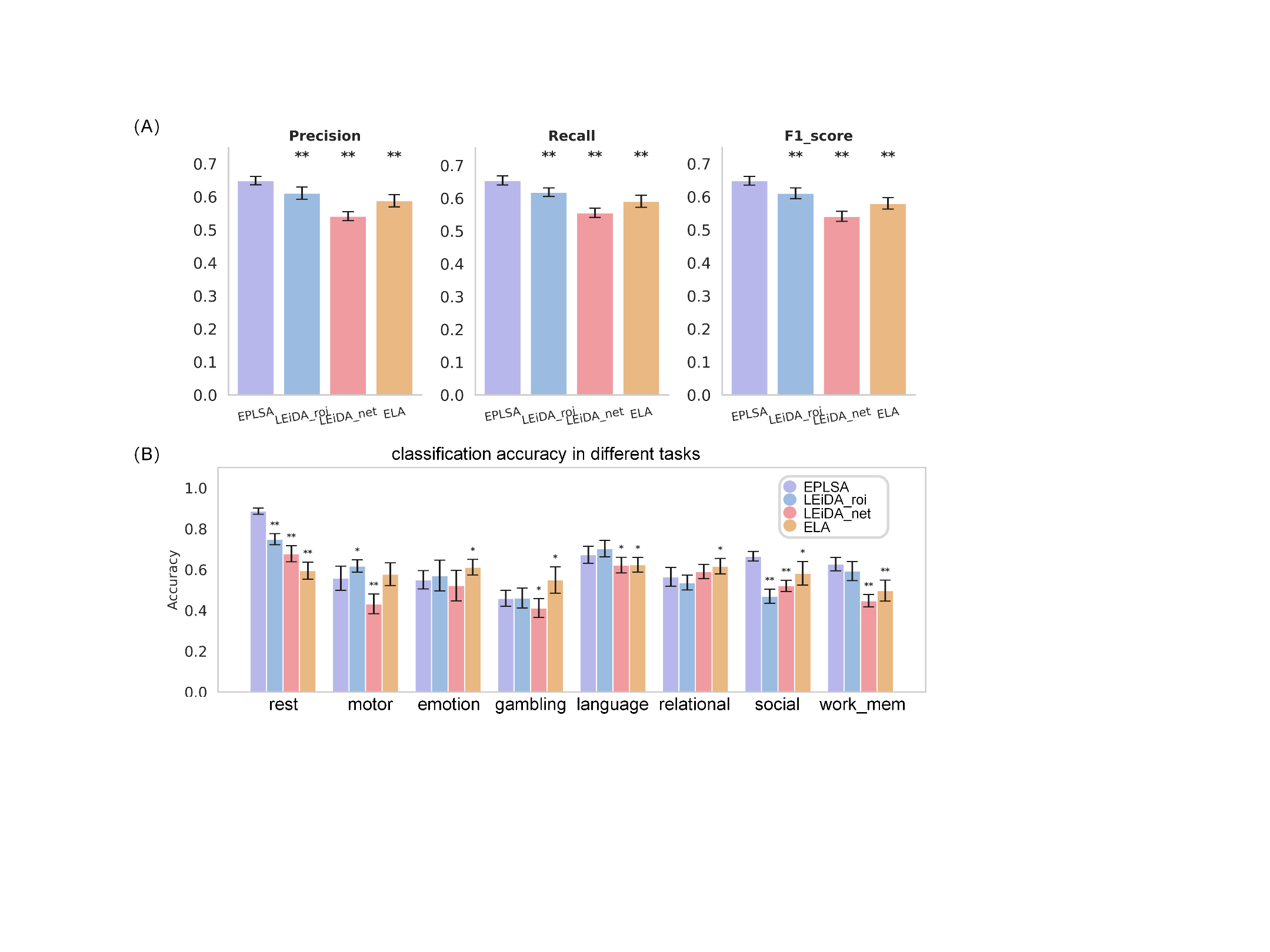


**Figure S9.** Between-group comparisons of brain state dwell times in sleep versus wakefulness.


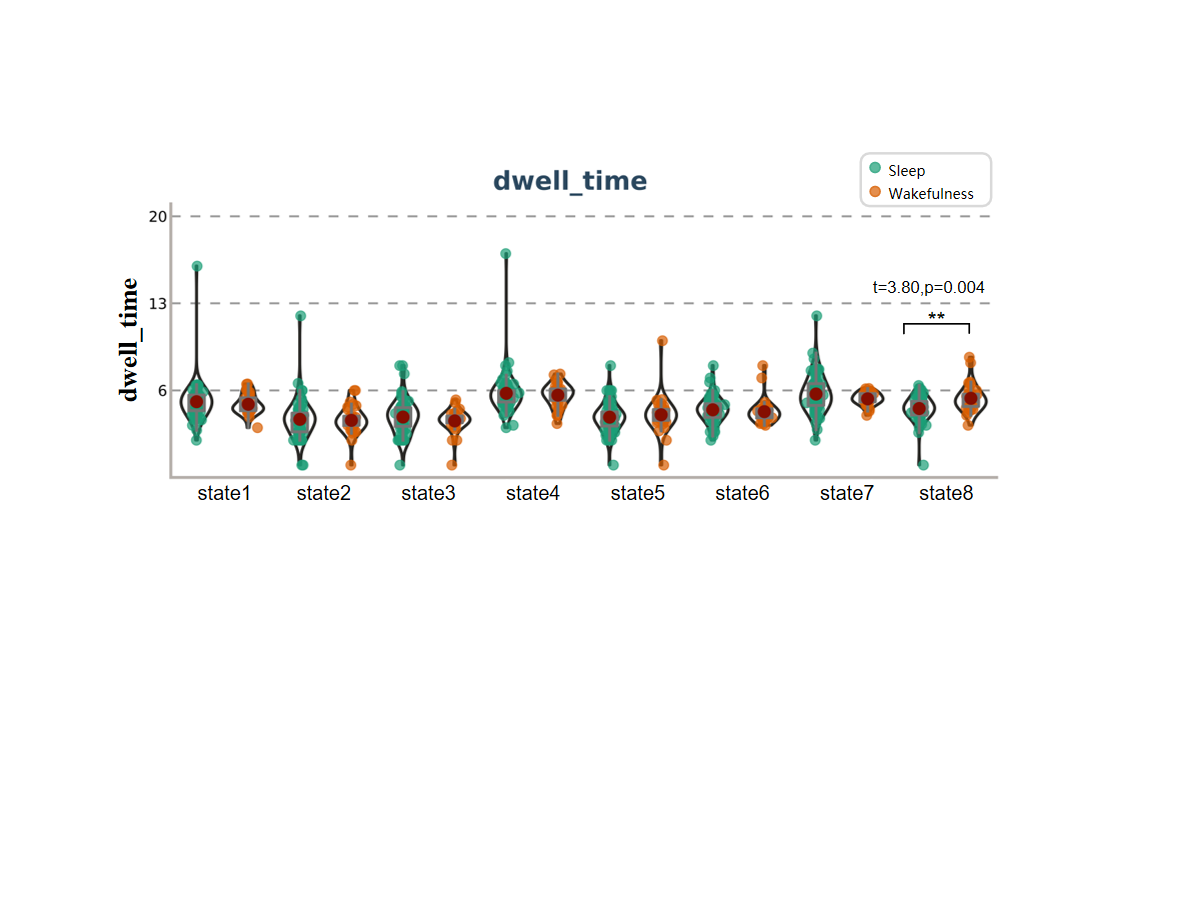


Table S1. Amyloid Positivity Thresholds by PET Tracer (see Su et al., 2019).

| Tracer | Uncorrected Threshold | PVE-Corrected Threshold (RSF) | Corresponding CL Value |
| --- | --- | --- | --- |
| [¹¹C]-PIB | \| MCBP ≤0.18; MCSUVR ≤1.31 \| \| --- \| | MCSUVR_RSF ≤1.42 | <16.4 (HC); ≥16.4 (AD) |
| [¹⁸F]-AV45 | MCSUVR ≤1.24 | MCSUVR_RSF ≤1.19 | <20.6 (HC); ≥20.6 (AD) |

Note: PVE = Partial Volume Effect; RSF = Regional Spread Function; CL conversion for PIB: Centiloid SUVR_RSF = 45.0 × PIB_3060_SUVR_RSF – 47.5; CL conversion for AV45: Centiloid SUVR_RSF = 53.6 × AV45_SUVR_RSF – 43.2.

Table S2. Definitions for different measurements.

| Quantitative measurements | Definition |
| --- | --- |
| Accuracy | $\frac{TP+TN}{TP+FP+TN+FN}$ |
| Recall | $\frac{TP}{TP+FN}$ |
| Precision | $\frac{TP}{TP+FP}$ |
| F1-score | $\frac{2TP}{TP+TP+FP+FN}$ |

Note: TP is the number of positive subjects that are predicted correctly. FN is the number of negative subjects that are predicted incorrectly, and similarly, TN and FP are the numbers of their corresponding subjects, respectively.

Table S3. The length of timeseries and MNR value in methods of rest and seven tasks.

| task | Length of timeseries | EPLSA | LEiDA_roi | LEiDA_net | ELA |
| --- | --- | --- | --- | --- | --- |
| Rest | 1200 | **0.6776** | 0.6689 | 0.6564 | 0.6062 |
| Working memory | 405 | **0.6096** | 0.5912 | 0.5905 | 0.5684 |
| Language | 316 | 0.5049 | **0.5859** | 0.5588 | 0.4908 |
| Motor | 283 | 0.5506 | **0.5643** | 0.5482 | 0.5454 |
| Social | 274 | **0.5998** | 0.5638 | 0.5529 | 0.5554 |
| Gambling | 253 | **0.6005** | 0.5692 | 0.5654 | 0.5665 |
| Relational | 232 | **0.5959** | 0.5560 | 0.5698 | 0.5460 |
| Emotion | 176 | 0.5341 | **0.5396** | 0.5298 | 0.5286 |

Note: The result of LEiDA_roi and LEiDA_net is the maximum value in the case of K = 2 – 10

***Reference***

1. Ezaki, T., Watanabe, T., Ohzeki, M., & Masuda, N. (2017). Energy landscape analysis of neuroimaging data. Philosophical Transactions of the Royal Society A, 375, 20160287. <https://doi.org/10.1098/rsta.2016.0287>
2. LaMontagne, P. J., Benzinger, T. L., Morris, J. C., Keefe, S., Hornbeck, R., Xiong, C., ... & Marcus, D. (2019). OASIS-3: longitudinal neuroimaging, clinical, and cognitive dataset for normal aging and Alzheimer disease. medrxiv, 2019-12.
3. Su, Y., Flores, S., Wang, G., Hornbeck, R. C., Speidel, B., Joseph‐Mathurin, N., ... & Benzinger, T. L. (2019). Comparison of Pittsburgh compound B and florbetapir in cross‐sectional and longitudinal studies. Alzheimer's & Dementia: Diagnosis, Assessment & Disease Monitoring, 11(1), 180-190.
